# Impact of immunosuppressive therapy and clinical risk factors on the prevalence of low bone mineral density in rheumatic diseases

**DOI:** 10.3389/fmed.2026.1897856

**Published:** 2026-09-14

**Authors:** Jan-Michael Brandt, Margret Patecki, Torsten Witte, Kai Schmidt-Ott, Vega Gödecke, Stefanie Hillmer

**Affiliations:** 1 Department of Rheumatology and Immunology, Hannover Medical School, Hannover, Germany; 2 Department of Nephrology and Hypertension, Hannover Medical School, Hannover, Germany; 3 Center for Renal, Hypertensive and Metabolic Disorders (ZNHS), Hannover, Germany

**Keywords:** bone mineral density, immunosuppressive agents, osteopenia, osteoporosis, prevalence, rheumatic diseases, risk factors

## Abstract

**Background:**

Osteoporosis is a severe comorbidity in inflammatory rheumatic diseases, impacting patient quality of life and healthcare costs. While general risk factors are known, the specific influence of various immunosuppressive therapies across different disease entities requires further characterization. This study aimed to determine the prevalence of low bone mineral density (BMD), identify clinical risk factors, and assess medication-associated factors in a large university cohort.

**Methods:**

A cross-sectional study of 704 adult patients receiving care at the specialized Rheumatology Outpatient Clinic of the Hannover Medical School was conducted. Data on lifestyle, medical history, and medication were collected via specialized questionnaires and electronic records. Low BMD was defined as a T-score ≤ −1.0. Statistical analyses included Pearson's chi-squared, Welch t-tests, and Wilcoxon rank-sum tests.

**Results:**

The prevalence of low BMD was 56%. Rheumatoid arthritis (RA) (65.8%) and systemic lupus erythematosus (SLE) (60.2%) showed the highest rates. Beyond age, female gender, and low BMI, alcohol consumption was significantly associated with low BMD across several diseases (RA: *p* = 0.009; SLE: *p* < 0.001; Sjögren's syndrome: *p* = 0.014). Regarding therapy, use of conventional synthetic DMARDs (csDMARDs) was associated with higher low BMD prevalence in RA (*p* = 0.005), specifically hydroxychloroquine (*p* = 0.016). Mycophenolate mofetil in SLE (*p* = 0.037) and hydroxychloroquine in other connective tissue diseases (*p* = 0.027) were also significant factors.

**Conclusion:**

Patients with rheumatic diseases exhibit a substantial and clinically relevant prevalence of low BMD. Our findings highlight an association between alcohol consumption and low BMD. The association of specific csDMARDs likely reflects higher cumulative disease severity, necessitating intensified bone density monitoring in these treatment groups.

## 1 Introduction

In Germany, approximately 5.6 million individuals are affected by osteoporosis, with a significantly higher prevalence in women over 50 years old (22.6%) than in men (6.6%) (1). The prevalence of osteoporosis in the overall population is estimated to be 6.1%, in the over 50-year-old group even 14% (2). Approximately 9 million osteoporotic fractures occur worldwide each year, with the highest incidence in Europe at approximately 35% (3), with a high impact on healthcare costs (4). Osteoporosis has a greater impact on quality of life (measured in disability adjusted life years) than most cancers and rheumatoid arthritis (RA) in European countries (3). Previously identified risk factors for the development of osteoporosis include advanced age, female gender, low body weight, smoking, and endocrine disorders (5–10).

Patients with inflammatory rheumatic diseases are at an increased risk for bone loss and fractures (11–13). Inflammatory rheumatic diseases affect approximately 1.5 million people in Germany, or about 2% of the adult population (14). RA is the most common inflammatory rheumatic disease (15). Approximately 30%–50% of patients with RA develop osteoporosis (16). The development of osteoporosis is determined by the severity and duration of RA, as well as the person's age and gender (17). Patients with RA have an increased risk of fracture, approximately 1.5 times higher than the general population (18), most noticeable in female RA patients under the age of 50 years (19). Glucocorticoid-induced osteoporosis is the most common form of secondary osteoporosis, and even low-dose prednisolone therapy of 2.5 mg daily can increase the risk of hip and vertebral fractures (20). Glucocorticoid therapy, as it is often used in rheumatic diseases, is therefore an additional risk factor for osteoporosis in these patients (16). On the other hand, cortisone is essential to suppress inflammation by regulating the inflammatory mediators, particularly interleukin (IL)-1, IL-6, and tumor necrosis factor-alpha (TNF-α). These mediators do not only act at the site of inflammation, but also lead to bone resorption (16).

The aim of the study is to provide a comprehensive overview of general and treatment-associated risk factors for the development of osteoporosis in this high-risk population. We examined the potential effect of individual rheumatic therapies on low BMD prevalence and reviewed known risk factors in patients with a rheumatic diagnosis. The extent to which the risk of developing osteoporosis differs according to disease entity and treatment modality has been studied little to date. Our analysis addresses this point and describes the association between lifestyle factors, underlying disease and drug therapy and the development of low BMD in a large university patient cohort. Understanding the potential risk factors and therapeutic effects will assist in the management of at-risk patients.

## 2 Methods

### 2.1 Study population

During a predefined prospective data collection period between March 2021 and November 2021, all consecutive adult patients (≥18 years) receiving care at the Rheumatology Outpatient Clinic of the Hannover Medical School were invited to participate in the study. The study questionnaire was integrated into the routine clinical registration process and handed to the patients upon arrival. In total, 1845 patients were approached in this manner. Access to this specialized tertiary care clinic requires prior medical evaluation, including a documented suspected diagnosis or referral from a primary care physician or specialist, accompanied by recent laboratory results. Consequently, the invited individuals represented a clinically pre-selected cohort. The study population consisted of individuals with either an available dual-energy x-ray absorptiometry (DXA) measurement at our institution or a previous DXA assessment outside the MHH. A total of 704 participants were enrolled in the study after providing written informed consent. For 352 patients, DXA measurements were available from our institution, including exact T-scores. A further 352 patients reported having undergone DXA assessment outside the MHH; for these patients, no external DXA reports were available, and information on osteoporosis was based on patient-reported medical history. The clinical assessment for the study was performed during the first outpatient visit within the study period. All rheumatological diagnoses were established by board-certified rheumatologists and were confirmed according to established international classification criteria, including the 2010 ACR/EULAR criteria for rheumatoid arthritis (RA), 2019 EULAR/ACR criteria for systemic lupus erythematosus (SLE), and ASAS criteria for spondyloarthritis (SpA). Sjögren's syndrome was classified according to the 2016 ACR/EULAR classification criteria, while vasculitides were classified according to the Chapel Hill Consensus Conference definitions and 2022 ACR/EULAR classification criteria.

### 2.2 Questionnaire development

The study utilized a custom-designed, self-administered questionnaire focused on capturing detailed patient history related to osteoporosis risk factors and medication use. This instrument was developed by a team of clinical experts including two experienced rheumatologists (one with a specialization in nephrology) and a nephrologist with expertise in osteology (MP, VG, SH). The development process ensured that the content was highly relevant to the intersection of rheumatic disease, immunosuppressive therapy, and bone health, and was rigorously guided by the established osteoporosis guidelines of the German-speaking scientific osteological societies (9), ensuring high content validity for clinical risk factors.

To ensure clarity, comprehensibility, and feasibility in a clinical setting, the questionnaire underwent an initial pilot phase during regular outpatient clinic hours prior to the study launch. During this phase, the questionnaire was distributed by the attending physicians of the department. Feedback regarding terminology, flow, and completion time was collected informally from both patients and the administering physicians, and subsequently reviewed collaboratively by the core team. As the questionnaire primarily assesses objective clinical facts (e.g., specific diagnoses, medication use) based on established guidelines, it demonstrated high face validity and required no significant structural adjustments. Given its nature as a clinical anamnesis tool rather than a psychometric scale, a formal psychometric validation process was not conducted. An English translation of the final questionnaire used in this study is provided in Supplementary Material S1.

### 2.3 Data collection

All patients completed a questionnaire regarding their history of osteoporosis. Data collected via the questionnaire included lifestyle factors, personal medical history, and medication history. DXA results, laboratory parameters, and current daily medication dose were exclusively extracted from electronic patient records. They were requested to provide their height and weight; body mass index (BMI) was then calculated by dividing the weight by the square of the height. Furthermore, we asked about osteoporosis risk factors as defined by the umbrella organization of the German-speaking scientific osteological societies in their osteoporosis guideline (9). For the lifestyle survey, data about the weekly frequency of sports activities, completion of 30 min of exercise daily, daily intake of coffee/tea and cigarettes, and weekly consumption of alcohol was collected. Alcohol consumption was assessed as a continuous variable (glasses per week) rather than a binary categorization, allowing for a more nuanced analysis of its association with bone mineral density (BMD). We inquired about atraumatic fractures and vertebral fractures, bone pain and parental femoral neck fractures.

The survey also included pre-existing conditions such as Cushing's syndrome, primary hyperparathyroidism, growth hormone deficiency due to pituitary insufficiency, subclinical and manifest hyperthyroidism, type I diabetes mellitus, type II diabetes mellitus, celiac disease, Billroth II gastric resection or gastrectomy, epilepsy, depression, schizophrenia, stroke, Alzheimer's disease, Parkinson's disease, cardiac insufficiency, renal insufficiency and monoclonal gammopathy of undetermined significance (MGUS).

We asked about the medication history, especially regarding treatment of prostate cancer, breast cancer or epilepsy, as well as intake of psychotropic drugs, prednisolone, glitazone, tranquilizers, sleeping pills, analgesics, or proton pump inhibitors (PPIs).

When available, the following laboratory parameters were evaluated: calcium, C-reactive protein (CRP), serum creatinine (sCr), estimated glomerular filtration rate (eGFR), alkaline phosphatase (ALP), erythrocyte sedimentation rate (ESR) 1 h/2 h, parathyroid hormone (PTH), and 25-hydroxyvitamin D (25(OH)D). For patients with internal DXA measurements, we evaluated the L1-L4 vertebrae, left/right femoral neck, and femur T-scores.

We have defined 9 patient subgroups based on the underlying disease. These include RA, systemic lupus erythematosus (SLE), Sjögren's syndrome (SjS), spondyloarthritis (SpA), other connective tissue disease (CTDs) (excluding RA, SLE and SjS), vasculitis, autoinflammatory syndrome, other rheumatic diseases (including polymyalgia rheumatica (*n* = 18), sarcoidosis (*n* = 13) and IgG4-related disease (*n* = 7), remaining cases (*n* ≤ 2 each) of retroperitoneal fibrosis, secondary antibody deficiency, undifferentiated arthritis, chronic recurrent multifocal osteomyelitis, eosinophilic fasciitis, polyglandular autoimmune syndrome, relapsing polychondritis and Schnitzler's syndrome) and cases that had not yet received a final diagnosis or remained undiagnosed at the time of assessment, despite presenting with symptoms requiring specialized rheumatological workup (patients in this group were subsequently classified as ‘undiagnosed’). The ‘other rheumatic diseases’ group was aggregated due to low individual case numbers, representing a relevant clinical patient mix.

The use of glucocorticoids and immunosuppressants was recorded; we divided the latter into the drug groups csDMARDs [hydroxychloroquine (HCQ), Azathioprine, mycophenolate mofetil (MMF), methotrexate (MTX), Leflunomide, Sulfasalazine], tsDMARDs (Apremilast, Baricitinib, Upadacitinib), bDMARDs [TNF-α inhibitors, Interleukin receptor antagonists (IL inhibitors) and monoclonal antibodies], TNF-α inhibitors (Adalimumab, Certolizumab, Etanercept, Golimumab, Infliximab), IL inhibitors (Anakinra, Canakinumab, Tocilizumab, Secukinumab, Ixekizumab), monoclonal antibodies [Rituximab (RTX), Belimumab, Abatacept], calcineurin inhibitors (Tacrolimus, Ciclosporin), and immunoglobulins (Intratect®, Kiovig®, Privigen®, Octagam®, Gamunex®, Cuvitru®, Gammanorm®, Hizentra®, Cutaquig®, HyQvia®, Other).

### 2.4 Statistical analysis

We have grouped patients according to their specific treatment methods to analyze whether a medication has an impact on the prevalence of low BMD in certain disease groups. We compared patients with and without low BMD in the entire cohort and by categorizing underlying disease using questionnaires, laboratory tests, diagnostic procedures, and patient records. To determine whether there was a significant difference between the low BMD and no low BMD groups, Pearson's chi-squared test was performed on categorical data, with results presented as percentages and numbers. For interval scale data, the Welch *t*-test was applied to account for potentially unequal variances between groups, and the means ± standard deviations were presented. Fisher's exact test was used for small sample size. For ordinal scale data, the Wilcoxon rank-sum test was used, and the median was presented with interquartile range (IQR) of 25%–75%. The analyses were performed utilizing RStudio software (version 2023.12.1, Posit Software, PBC, Boston, MA). Results achieved statistical significance at a *p*-value of less than 0.05.

For this analysis, the primary outcome variable ‘low BMD’ was defined as a T-score ≤ −1.0; in patients without available DXA measurements, a history of osteoporosis was considered indicative of low BMD. This inclusive definition of low BMD was chosen to reflect the specific ‘at-risk’ population in inflammatory rheumatic diseases, where the fracture threshold often occurs at significantly higher BMD levels than in the general population. Chronic systemic inflammation and the frequent use of glucocorticoids are known to impair bone quality independently of the T-score. Furthermore, current clinical guidelines, such as those from the DVO (Dachverband Osteologie), recommend that therapeutic intervention and risk assessment should start in the osteopenic range if high-risk factors like rheumatoid arthritis or systemic lupus erythematosus are present (21).

## 3 Results

### 3.1 Demographics and prevalence of low BMD

The patient population consists of 576 (81.8%) females and 128 (18.2%) males. The proportion of women in the population increases with age. It reaches a peak in the 40–49 age group (85%) and then declines slightly. Overall, the proportion of women remains significantly higher for patients aged 30 or older. The number of women is higher in all disease subgroups: SjS (98.7%), SLE (88.5%), other connective tissue diseases (82.9%), rheumatoid arthritis (79.5%), spondyloarthritis (78.6%) and vasculitis (77.2%). The proportion of female patients was lower in the autoinflammatory group (61.5%). Except for autoinflammation (median age 51 years), the median age for each disease group is 57 years or higher (Table 1).

**Table 1:** Cohort demographics and prevalence of low bone mineral density (BMD).

Group	Total *N* (%)	Female *n* (%)	Median age [Range] (years)	Low BMD (T ≤ −1.0) *n* (%)
Total cohort	704 (100)	576 (81.8)	62 [18–87]	394 (56.0)
Age groups				
18–29 years	6 (0.9)	2 (33.3)	–	0 (0.0)
30–39 years	28 (4.0)	20 (71.4)	–	17 (60.7)
40–49 years	80 (11.4)	68 (85.0)	–	33 (41.3)
50–59 years	191 (27.1)	160 (83.8)	–	89 (46.6)
60–69 years	226 (32.1)	182 (80.5)	–	138 (61.1)
≥70 years	173 (24.6)	144 (83.2)	–	117 (67.6)
Diagnosis				
Rheumatoid arthritis	146 (20.7)	116 (79.5)	66 [29–87]	96 (65.8)
Systemic lupus erythematosus	113 (16.1)	100 (88.5)	57 [19–82]	68 (60.2)
Spondyloarthritis	126 (17.9)	99 (78.6)	60 [31–86]	58 (46.0)
Connective tissue disease^a^	82 (11.6)	68 (82.9)	58.5 [19–86]	47 (57.3)
Vasculitis	79 (11.2)	61 (77.2)	64 [18–82]	47 (59.5)
Sjögren's syndrome	79 (11.2)	78 (98.7)	63 [33–81]	47 (59.5)
Other rheumatic diseases	51 (7.2)	36 (70.6)	64 [45–86]	21 (41.2)
Autoinflammatory syndromes	13 (1.8)	8 (61.5)	51 [25–81]	7 (53.8)
Undiagnosed	15 (2.1)	10 (66.7)	62 [32–78]	3 (20.0)

Low BMD was defined as described in the Methods. Values are presented as absolute numbers (n) and percentages (%) or medians with ranges.

a Connective tissue diseases excluding SLE and SjS.

Of the 352 patients with internal DXA measurements, 181 (51.4%) were classified as having osteopenia and 62 (17.6%) as having manifest osteoporosis. A distribution of T-values is displayed (Table 2). A further 352 patients reported having undergone a DXA scan outside the MHH, with a total of 151 (42.9%) people reporting osteoporosis diagnosed as result.

**Table 2:** Distribution of T-scores and classification of bone mineral density*.

Classification	T-score range	*n* (%)	Mean T-score ± SD	Median [IQR]
Normal	>−1.0	109 (31.0)	−0.2 ± 0.7	−0.4 [−0.7 to 0.2]
Osteopenia	≤−1.0 and >−2.5	181 (51.4)	−1.7 ± 0.4	−1.8 [−2.0 to −1.4]
Manifest Osteoporosis	≤−2.5	62 (17.6)	−3.1 ± 0.4	−3.0 [−3.3 to −2.7]
Total		**352 (100)**	**−1.5 ± 1.1**	**−1.6 [−2.2 to −0.8]**

*Data refers to the subgroup of patients with available internal DXA measurements at Hannover Medical School. Classification follows WHO criteria, while the study’s primary endpoint “low BMD” encompasses both osteopenia and manifest osteoporosis. Bold values indicate the total cohort.

In our cohort, the prevalence of low BMD is 56% (394/704). In the 18–29 age group, nobody reported to have low BMD. The prevalence among individuals aged 30–39 is 60.7%. In the 40–49 age group, the prevalence drops to 41.3%, before rising continuously: In the 50–59 age group to 46.6%, in the 60–69 age group to 61.1%, and in the 70+ age group to 67.6%. The highest prevalence is seen for RA patients (65.8%), followed by SLE (60.2%), SjS (59.5%), vasculitis (59.5%) and other CTD patients (57.3%). A slightly lower prevalence of low BMD can be seen in patients with autoinflammatory syndromes (53.8%), SpA (46%) and other diagnoses group (41.2%) (Table 1).

### 3.2 Risk factors for low BMD in rheumatic and immunological diseases

The risk factor analysis for low BMD in the entire cohort indicates that women are affected by low BMD more frequently than men (*p* = 0.036). In addition, patients with low BMD are found to be significantly older (*p* < 0.001) and to have lower height (*p* < 0.001) and body weight (*p* < 0.001) than patients without. Coffee and tea do not appear to be relevant risk factors, whereas alcohol consumption (*p* < 0.001) and current smoking (*p* < 0.001) were significantly associated with low BMD. There is no significant association between reduced daily or weekly physical activity and a higher prevalence of low BMD. Vertebral and atraumatic fractures are more common in patients with low BMD (*p* < 0.001), with a slight, although not significant increase in the number of atraumatic fractures (*p* = 0.085). A family history of femoral neck fractures is not significantly associated with low BMD. Patients with low BMD report significantly more severe bone pain [4 (1–7) points on numeric rating scale (NRS)] compared to those without [3 (0–6) on NRS] (*p* = 0.042). Lower ALP (*p* = 0.004) values are associated with an increased prevalence of low BMD. However, we find no association between calcium or CRP values and increased prevalence of low BMD in the overall cohort. Since sCr values are also not associated with higher low BMD prevalence, the observed association with lower eGFR may be confounded by age and gender rather than reflecting renal function itself. Elevated ESR at both the 1-hour and 2-hour intervals (*p* = 0.035 and *p* = 0.012), and elevated PTH (*p* = 0.048) may indicate an increased likelihood of low BMD. Higher 25(OH)D levels (*p* < 0.001) are found in patients with low BMD. The low BMD group exhibits a higher prevalence of several preexisting conditions. However, only renal insufficiency (*p* = 0.041) is found to be statistically significant. A lower prevalence of type II diabetes is observed in the low BMD group (*p* = 0.024). The medication associated with an increased risk of low BMD includes prednisolone (*p* = 0.006), analgesics (*p* = 0.007) and PPI (*p* = 0.031) (Table 3; Supplementary Table S1).

**Table 3:** Clinical and lifestyle risk factors for low bone mineral density (BMD).

Parameter	No low BMD (*n* = 310)	Low BMD (*n* = 394)	*p*-value
Demographics			
Gender (Female), *n* (%)	243 (78.4)	333 (84.5)	**0**.**036**
Age, years (mean ± SD)	58.2 ± 12.3	62.9 ± 11.7	**<0**.**001**
Body Mass Index, kg/m^2^	28.1 ± 6.0	25.7 ± 5.6	**<0**.**001**
Height, cm	169.0 ± 9.2	166.5 ± 8.3	**<0**.**001**
Weight, kg	80.3 ± 18.3	71.5 ± 17.3	**<0**.**001**
Lifestyle			
Alcohol, glasses/week	0.9 ± 1.7	2.9 ± 1.9	**<0**.**001**
Cigarettes, n/day	1.7 ± 4.5	10.5 ± 6.6	**<0**.**001**
Exercise (≥30 min/day), *n* (%)	265 (85.5)	326 (82.7)	0.517
Clinical presentation			
Vertebral fractures, *n* (%)	15 (4.8)	79 (20.1)	**<0**.**001**
Atraumatic fractures, *n* (%)	22 (7.1)	70 (17.8)	**<0**.**001**
Bone pain (NRS 0–10), median [IQR]	3 [0–6]	4 [1–7]	**0**.**042**
Laboratory Values			
Calcium, mmol/l	2.4 ± 0.1	2.4 ± 0.1	0.755
CRP, mg/l	5.1 ± 9.8	3.9 ± 9.7	0.102
Serum Creatinine, µmol/l	79.5 ± 27.7	81.9 ± 36.1	0.317
eGFR, ml/min	79.8 ± 18.2	75.4 ± 19.7	**0**.**003**
Alkaline Phosphatase, U/l	83.2 ± 34.6	76.2 ± 26.0	**0**.**004**
ESR (1 h), mm	13.2 ± 12.9	15.4 ± 13.8	**0**.**035**
25-hydroxyvitamin D, µg/l	31.1 ± 13.2	37.0 ± 15.1	**<0**.**001**
Medication			
Prednisolone, *n* (%)	127 (41.0)	194 (49.2)	**0**.**006**
Analgesics, *n* (%)	26 (8.4)	57 (14.5)	**0**.**007**
Proton Pump Inhibitors, *n* (%)	118 (38.1)	174 (44.2)	**0**.**031**

Low BMD was defined as described in the Methods. Values are presented as mean ± SD, median [IQR], or *n* (%) as appropriate.

CRP, C-reactive protein; eGFR, estimated glomerular filtration rate; ESR, erythrocyte sedimentation rate; NRS, numeric rating scale. Bold *p*-values indicate statistically significant differences (*p* <0.05).

Since anti-osteoporotic medications are specifically indicated for bone loss, their use was exclusively observed in the group of patients with low BMD. Within this affected cohort (data available for *n* = 390), 137 patients (35.1%) reported a history of anti-osteoporotic treatment. The most frequently prescribed agents were bisphosphonates, predominantly alendronate (*n* = 74, 19.0%), ibandronate (*n* = 19, 4.9%), risedronate (*n* = 9, 2.3%), and zoledronate (*n* = 9, 2.3%). Regarding specific osteoporosis treatments, antiresorptive therapies were widely used across the cohort. In contrast, osteoanabolic therapy (i.e., teriparatide) was present in only a marginal fraction of patients (*n* = 6; 0.85%) and was consequently not further investigated in the comparative analyses due to limited statistical power. The RANKL-inhibitor denosumab had been used by 34 patients (8.7%). Regarding the current treatment status, only 76 out of 378 patients with available data (20.1%) were actively taking bone-specific therapeutics at the time of the survey, highlighting a potential gap in continuous osteological care. Given the specific relevance of osteoclast inhibition in erosive joint diseases, a subgroup analysis of patients with rheumatoid arthritis (RA) and low BMD revealed an even higher treatment frequency: 41.1% (39 out of 95) had a history of anti-osteoporotic treatment, while 27.8% (25 out of 90) were currently receiving therapy. A complete breakdown of anti-osteoporotic medication use across all disease subgroups is provided in Supplementary Table S2.

### 3.3 Risk factors for the rheumatic and immunological disease groups

In comparison to the entire group, we identified different risk factors for each disease group (Table 4, Supplementary Table S3, S4). In patients with RA, female gender (*p* = 0.004), older age (*p* = 0.002), smaller height (*p* = 0.005) and weight (*p* = 0.001), alcohol (*p* = 0.009) and cigarette (*p* = 0.001) consumption, more atraumatic (*p* = 0.008) and vertebral fractures (*p* < 0.001) and lower ALP (*p* = 0.005) are associated with low BMD. Furthermore, a lower CRP (*p* = 0.011) is observed. In patients with SLE, an increased prevalence is observed with older age (*p* = 0.007), lower weight (*p* = 0.002), alcohol (*p* < 0.001) and cigarette consumption (*p* = 0.039). In SpA, lower height (*p* = 0.001), cigarette consumption (*p* = 0.001), vertebral (*p* = 0.005) and atraumatic fractures (*p* = 0.008) are correlated. Again, a lower prevalence of type II diabetes is observed (*p* = 0.033). For other CTD, our findings indicate that a low consumption of tea (*p* = 0.026), lower weight (*p* = 0.009), alcohol (*p* = 0.015) and cigarette consumption (*p* = 0.003), a history of vertebral fracture (*p* = 0.002) and the use of analgesics (*p* = 0.017) appear to be associated with a higher prevalence of low BMD. For vasculitis, only lower eGFR (*p* = 0.049), cigarette consumption (*p* = 0.015) and higher ESR for 1 h (*p* = 0.046) are identified as potential risk factors. In patients with SjS, low BMD was significantly associated with alcohol consumption (*p* = 0.014), vertebral (*p* = 0.013) and lower ALP (*p* = 0.039). Given the small number of patients with autoinflammatory disease, only older age (*p* = 0.025) shows a significant increase in the prevalence of low BMD.

**Table 4:** Significant risk factors and medication associations for low BMD by disease subgroup.

Diagnosis group	Risk factor/Medication	No low BMD	Low BMD	*p*-value
Rheumatoid Arthritis				
	Gender (Female), *n* (%)	33/50 (66.0)	83/96 (86.5)	0.004
	Age, years (mean ± SD)	60.3 ± 11.8	66.7 ± 11.3	0.002
	BMI, kg/m^2^	27.6 ± 5.4	25.4 ± 5.7	0.025
	Alcohol, glasses/week	1.6 ± 2.8	3.2 ± 1.7	0.009
	Cigarettes, n/day	2.5 ± 4.5	9.5 ± 6.1	0.001
	Vertebral fractures, *n* (%)	1/50 (2.0)	25/96 (26.0)	< 0.001
	csDMARDs, *n* (%)	16/50 (32.0)	54/96 (56.3)	0.005
	Hydroxychloroquine, *n* (%)	0/50 (0.0)	11/96 (11.5)	0.016
Systemic Lupus Erythematosus				
	Age, years (mean ± SD)	52.7 ± 11.4	58.8 ± 11.7	0.007
	Alcohol, glasses/week	1.0 ± 1.5	2.6 ± 1.0	< 0.001
	BMI, kg/m²	27.9 ± 6.3	25.1 ± 5.0	0.013
	Mycophenolate mofetil, *n* (%)	10/45 (22.2)	28/68 (41.2)	0.037
Spondyloarthritis				
	BMI, kg/m²	30.0 ± 5.7	26.4 ± 5.7	0.001
	Cigarettes, n/day	0.8 ± 2.3	12.5 ± 8.0	0.001
	Atraumatic fractures, *n* (%)	7/68 (10.3)	16/58 (27.6)	0.008
Other CTDs^a^				
	Alcohol, glasses/week	0.8 ± 1.3	3.4 ± 2.1	0.015
	Hydroxychloroquine, *n* (%)	5/35 (14.3)	17/47 (36.2)	0.027
	Calcineurin inhibitors, *n* (%)	5/35 (14.3)	0/47 (0.0)	0.012
Sjögren's syndrome				
	Alcohol, glasses/week	0.2 ± 0.7	3.2 ± 2.3	0.014
	Vertebral fractures, *n* (%)	2/32 (6.3)	13/47 (27.7)	0.013
Vasculitis				
	eGFR, ml/min	79.1 ± 24.7	68.1 ± 21.4	0.049
	Cigarettes, n/day	3.3 ± 6.1	13.9 ± 5.0	0.015

Low BMD was defined as described in the Methods. Values are presented as mean ± SD or n/N (%).

BMI, body mass index; csDMARDs, conventional synthetic disease-modifying antirheumatic drugs; CTD, connective tissue disease; eGFR, estimated glomerular filtration rate.

a Connective tissue diseases excluding SLE and SjS.

### 3.4 Immunosuppressants as potential risk factors for low BMD

For all disease subgroups and immunosuppressants or immunoglobulins with sufficient data for analysis, we examined whether the use of specific medications is associated with the prevalence of low BMD. Sample sizes of 2 or less are not included. Our analysis revealed several significant associations between immunosuppressant use and low BMD across various rheumatic diseases. In RA patients, those with low BMD had a significantly higher frequency of csDMARD use compared to those without (56.3% vs. 32.0%, *p* = 0.005). Specifically, the use of HCQ was observed exclusively in the low BMD group (11.5% vs. 0%, *p* = 0.016). While the use of bDMARDs was higher in patients with low BMD (61.5% vs. 52.0%), this difference was not statistically significant (*p* = 0.272) (Figure 1).

**Figure 1:**
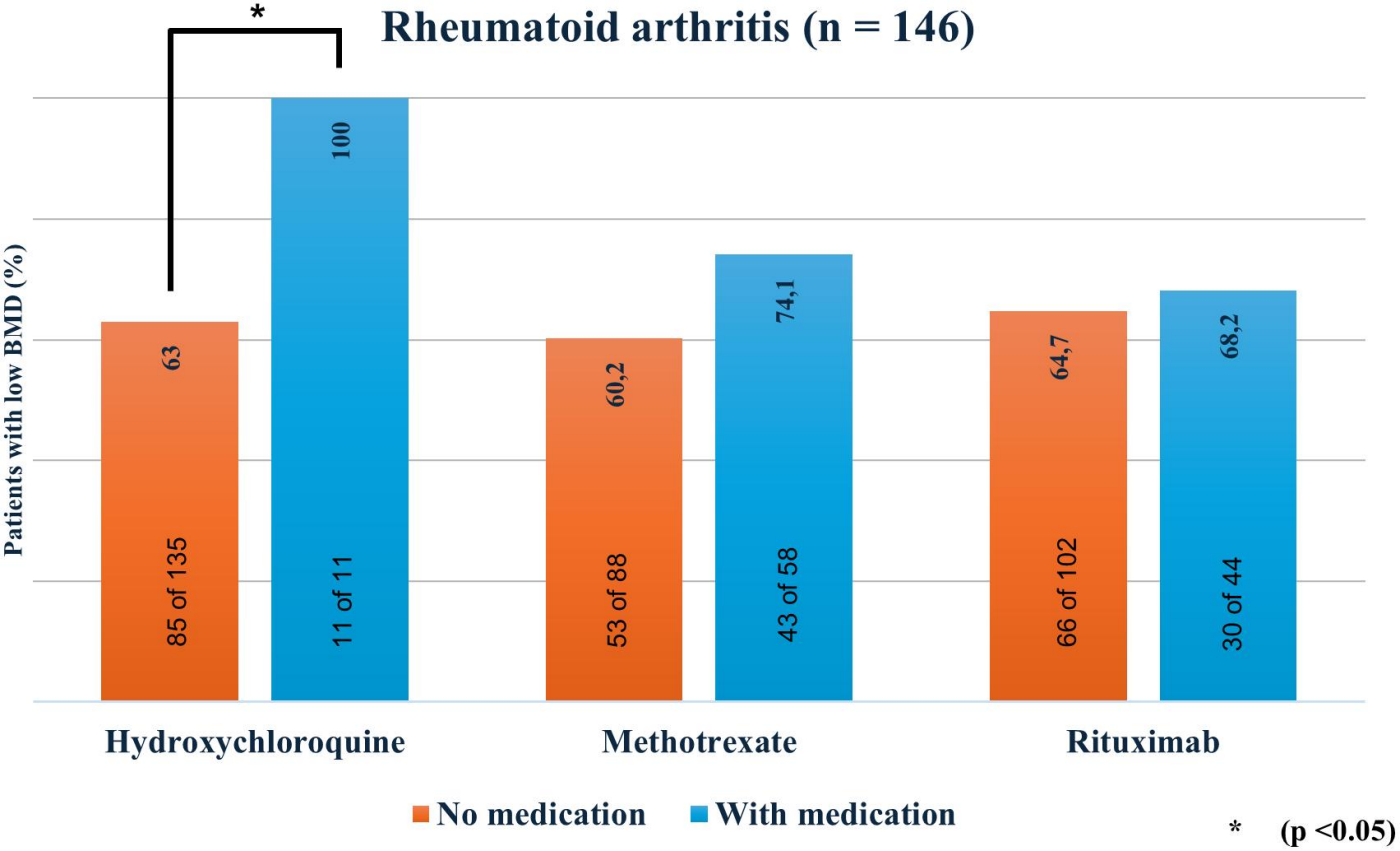
Prevalence of low bone mineral density (BMD) according to specific immunosuppressive therapies in rheumatoid arthritis (*n* = 146). The bar chart illustrates the percentage of patients with low BMD compared between those receiving specific medications (blue bars) and those not receiving them (orange bars). In the RA cohort, hydroxychloroquine (HCQ) use was significantly associated with low BMD (100% vs. 63%, *p* = 0.016). Numbers within the bars indicate the absolute number of affected patients and the total subgroup size. Significant differences are marked with an asterisk (*p < 0.05).

For SLE patients, a significantly higher proportion of the low BMD group was treated with MMF compared to the non-low BMD group (41.2% vs. 22.2%, *p* = 0.037). The use of csDMARDs in general and bDMARDs was also higher in the low BMD group, but these differences did not reach statistical significance (Figure 2).

**Figure 2:**
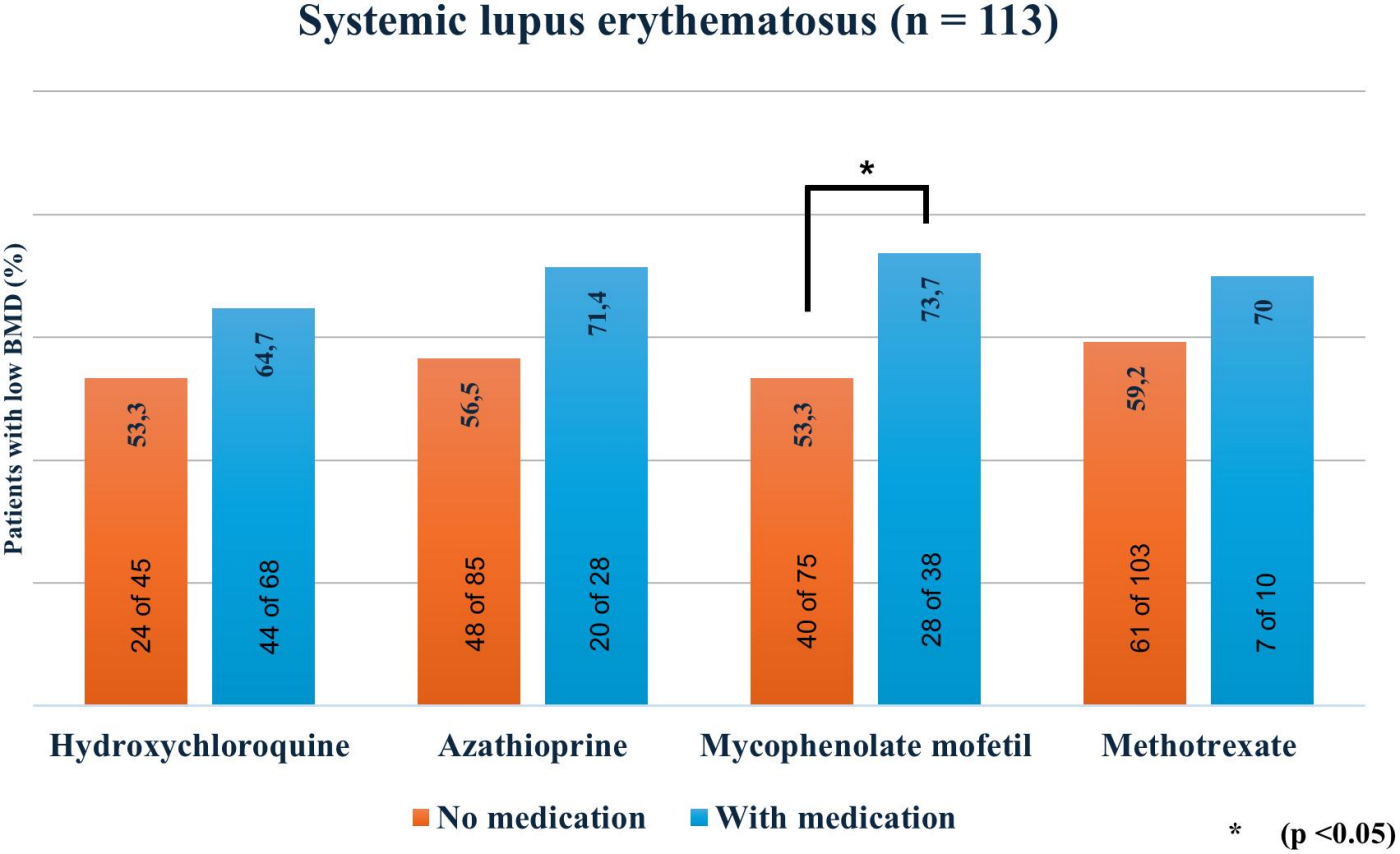
Prevalence of low bone mineral density (BMD) according to specific immunosuppressive therapies in systemic lupus erythematosus (*n* = 113). The bar chart illustrates the percentage of patients with low BMD compared between those receiving specific medications (blue bars) and those not receiving them (orange bars). In the SLE cohort, a significant association was identified for mycophenolate mofetil (MMF), where 73.7% of treated patients exhibited low BMD compared to 53.3% of untreated patients (*p* = 0.037). Numbers within the bars indicate the absolute number of affected patients and the total subgroup size. Significant differences are marked with an asterisk (*p < 0.05).

In the SpA group, while not statistically significant, there was a trend towards increased use of bDMARDs in patients with low BMD (65.5% vs. 48.5%, *p* = 0.055). The use of any immunosuppressant was higher in patients with low BMD (87.9% vs. 75.0%), but this difference was not statistically significant (*p* = 0.065). Similarly, patients with other CTDs showed a significantly higher prevalence of HCQ use in the low BMD group (36.2% vs. 14.3%, *p* = 0.027). Interestingly, no patients with low BMD were using calcineurin inhibitors compared to 14.3% in the non-low BMD group (*p* = 0.012) (Figure 3).

**Figure 3:**
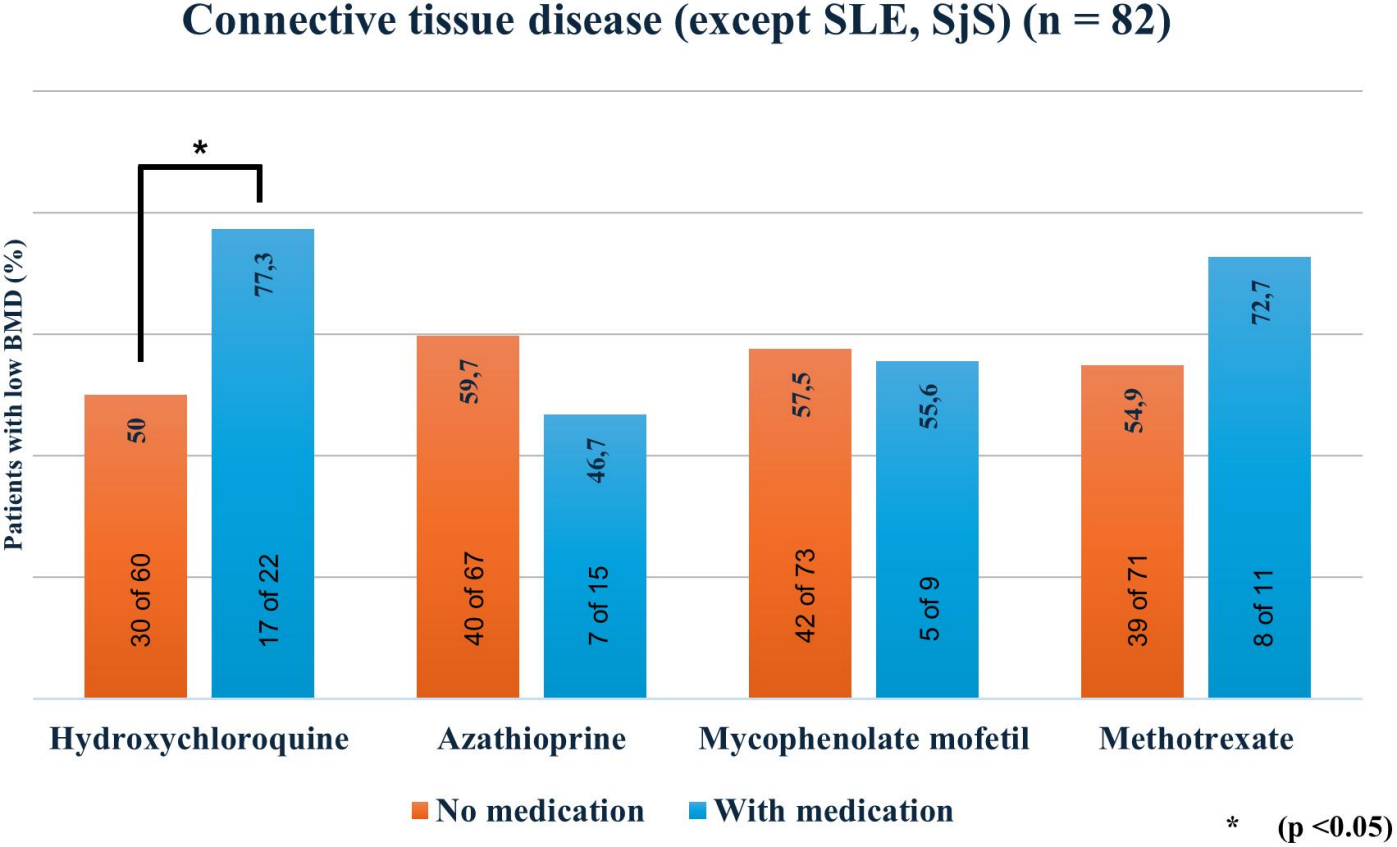
Prevalence of low bone mineral density (BMD) according to specific immunosuppressive therapies in other connective tissue diseases (except SLE and SjS, *n* = 82). The bar chart illustrates the percentage of patients with low BMD compared between those receiving specific medications (blue bars) and those not receiving them (orange bars). In the other CTD cohort, Hydroxychloroquine (HCQ) use was significantly associated with an increased prevalence of low BMD (77.3% vs. 50.0%, *p* = 0.027). Numbers within the bars indicate the absolute number of affected patients and the total subgroup size. Significant differences are marked with an asterisk (*p < 0.05).

For vasculitis, SjS, and autoinflammatory diseases, although there were often higher prevalences of medication use in the low BMD groups, these differences did not reach statistical significance. In the other diseases category, patients with low BMD had significantly higher use of csDMARDs (71.4% vs. 33.3%, *p* = 0.007), especially MTX (66.7% vs. 13.3%, *p* < 0.001), or any immunosuppressant (71.4% vs. 36.7%, *p* = 0.015) (Supplementary Table S5).

## 4 Discussion

In our cohort, the prevalence of patients with low BMD is 56%. Because the study population consisted of patients who underwent DXA assessment, the observed prevalence may not be representative of the broader rheumatology outpatient population. There are comparative values for Germany that indicate a prevalence of osteoporosis of 6.1% in the total population and 14% in individuals over 50 years of age (2).

In this study, the primary outcome ‘low BMD’ was defined using a T-score threshold of ≤−1.0, thereby encompassing both manifest osteoporosis and osteopenia. While this definition leads to a higher reported prevalence of 56% compared to studies using the stricter WHO threshold of ≤−2.5, we consider this inclusive approach clinically justified for several reasons.

First, patients with inflammatory rheumatic diseases are known to have an increased risk of fragility fractures even at higher BMD levels than the general population. Factors such as chronic systemic inflammation (e.g., IL-1, IL-6, TNF-α) and the frequent use of glucocorticoids significantly impair bone quality independent of the T-score. Second, current clinical guidelines, such as those from the DVO (Dachverband Osteologie), emphasize that therapeutic intervention and risk assessment should start in the osteopenic range if additional high-risk factors—such as rheumatoid arthritis or systemic lupus erythematosus—are present (21).

By including osteopenia, our data more accurately reflect the ‘at-risk’ population within a rheumatological cohort where bone loss is often accelerated and the fracture threshold is lower. However, it must be acknowledged that this broader definition limits direct comparability with epidemiological studies focusing solely on manifest osteoporosis, which typically report lower prevalence rates in the general population.

In patients with rheumatic diseases the prevalence of osteoporosis has been shown to be significantly higher. The reported results vary between the underlying diseases and between studies. RA with prevalence of up to 90.8% (22) and SLE with 25%–74% (16, 23) have the highest reported rates. Not without reason these diseases are considered risk factors for the development of osteoporosis and bone fractures (9, 12, 24–27). In our cohort RA (65.8%) and SLE (60.2%) patients also have the highest prevalence, but our values do not reach the maximum reported data. One possible reason for that might be that we included younger patients and men despite most studies focusing on postmenopausal women.

SpA is also considered as risk factor for osteoporosis (9, 11, 28). Various studies have reported a prevalence of 11.7% to 34.4% in axial SpA (29). In our cohort, 46% of SpA patients had low BMD, which is one of the lowest prevalences in our subgroups but still above the reported range.

Interestingly, patients with vasculitis and SjS have high low BMD prevalence in our study cohort. A study on vasculitis revealed a prevalence of 16% for giant cell arteritis (30) and another study indicated a prevalence of 14.1% for anti-neutrophil cytoplasmatic antibody (ANCA)-associated vasculitis (31). In contrast, the prevalence of low BMD among our cohort was found to be 59.5% for the vasculitis group. Our study also shows a prevalence of 59.5% for low BMD in patients with Sjögren's syndrome, compared to a prevalence of 18.5% for osteoporosis in a recent study (32).

The general risk factors for the development of osteoporosis already defined by the umbrella organization of German-speaking scientific osteological societies in its guideline on the prevention, diagnosis, and treatment of osteoporosis in postmenopausal women and men aged 50 and over (9) were largely confirmed in our study. Studies have shown a strong correlation between low BMI and increased fractures (10). In our study, patients with low BMD had a significantly lower BMI (25.7 ± 5.6) compared to patients without low BMD (28.1 ± 6) (*p* < 0.001).

A notable finding in our study is the significant association between alcohol consumption and low BMD. In the overall cohort, patients with low BMD reported higher alcohol consumption (2.9 ± 1.9 glasses/week) compared to those without low BMD (0.9 ± 1.7 glasses/week, *p* < 0.001). This trend was consistent across several disease subgroups, with statistically significant differences observed in RA (3.2 ± 1.7 vs. 1.6 ± 2.8 glasses/week, *p* = 0.009), SLE (2.6 ± 1.0 vs. 1.0 ± 1.5 glasses/week, *p* < 0.001), other CTDs (3.4 ± 2.1 vs. 0.8 ± 1.3 glasses/week, *p* = 0.015), and SjS (3.2 ± 2.3 vs. 0.2 ± 0.7 glasses/week, *p* = 0.014). This finding contrasts with some previous research on alcohol's effects in rheumatic conditions. For instance, several studies have reported potential protective effects of moderate alcohol consumption against RA development and disease activity (33, 34). In SLE, a 39% reduction in SLE risk among women consuming ≥5 g/day of alcohol was observed (35). However, the relationship between alcohol and bone health in rheumatic diseases remains complex and controversial. Some research suggests that excessive alcohol intake may negatively impact bone health and increase fracture risk (36).

While the reported average consumption was remarkably low, the high statistical significance across multiple disease entities (RA, SLE, SjS) suggests that even low-level alcohol intake may have a detrimental effect in the context of chronic inflammation. However, we must consider a potential ‘underreporting bias’ common in clinical questionnaires. The remarkably low average consumption levels reported by our patients suggest a potential 'social desirability bias’ or significant underreporting in a clinical setting. Furthermore, a 'sick-quitter effect’ might influence the data, as patients with more severe disease or those taking complex medication regimens, such as MTX, may have already reduced their alcohol intake significantly due to medical advice or potential side effects. This shift in behavior could paradoxically skew the statistical association. In a population already compromised by glucocorticoids and systemic cytokines, the observed association between alcohol consumption and low BMD may be particularly relevant in patients with chronic inflammatory disease.

Despite these potential biases, it remains plausible that even low amounts of alcohol may be associated with poorer bone health. In a population already compromised by chronic systemic inflammation and frequent glucocorticoid use, the physiological reserve of bone-forming cells is limited, making the skeleton more vulnerable to lifestyle-related risks. Consequently, the high prevalence of 56% low BMD in our cohort highlights the need for lifestyle counseling regarding alcohol intake. Future studies should utilize validated screening tools, such as the AUDIT-C, to minimize recall bias and better characterize the dose-response relationship in rheumatic cohorts.

Interestingly, our study found significantly higher 25(OH)D levels in patients with low BMD compared to those without (37 ± 15.1 µg/L vs. 31.1 µg/L ± 13.2, *p* < 0.001). This may reflect a treatment effect within the tertiary referral setting. Following an initial diagnosis of osteopenia or osteoporosis, these patients typically receive standardized supplementation as part of their specialized care. This underscores the adherence to clinical guidelines in our cohort but also highlights that a single vitamin D measurement may not reflect the long-term nutritional or metabolic bone status prior to diagnosis.

The present study offers significant insights into the association between anti-rheumatic therapies and the risk of low BMD in patients diagnosed with various rheumatic diseases. The findings underscore the complex interplay between disease-modifying treatments and bone health in these patient populations.

For RA patients, our results suggest that the use of csDMARDs may be associated with a higher prevalence of low BMD (56.3% vs. 32.0%, *p* = 0.005). This finding aligns with a recent study, which reported a 40% increased risk of major osteoporotic fractures in RA patients compared to those without RA, with a similar risk observed for csDMARD user (37). Other studies have not found a further increased risk of fractures in patients with RA on csDMARDs (38). Our data showed no significant protective effects of bDMARDs or tsDMARDs against low BMD in RA, which contrasts with some previous studies suggesting potential bone-preserving properties of these agents (39, 40).

In SLE patients, we found a significant association between MMF use and low BMD (41.2% vs. 22.2%, *p* = 0.037) despite recent findings of positive effects of MMF on maintaining normal osteoclastogenesis in SLE patients (41). This finding highlights the need for careful bone health monitoring in SLE patients. We found no further therapies associated with an increased or reduced risk of low BMD in SLE.

In patients with SjS, the use of HCQ seems to have no impact on the prevalence of low BMD and therefore does not confirm the hypothesis that HCQ use may prevent bone loss in SjS as shown elsewhere (42).

In other CTDs, the higher use of HCQ in patients with low BMD (36.2% vs. 14.3%, *p* = 0.027) was also unexpected. Interestingly, we found no patients with low BMD using calcineurin inhibitors in this group, compared to 14.3% in the non-low BMD group (*p* = 0.012). Previous studies have suggested that calcineurin inhibitors may contribute to bone loss and increase fracture risk in transplant patients (43). However, it's important to interpret our findings with caution due to the limited sample size in our study.

For autoinflammatory diseases, SpA and vasculitis we did not find treatments associated with low BMD prevalence.

For diseases that could not be assigned to the predefined groups (‘other’), the use of csDMARDs showed a significant increase in low BMD prevalence (71.4% vs. 33.3%, *p* = 0.007), particularly the use of MTX (66.7% vs. 13.3%, *p* < 0.001). These findings align with concerns raised about MTX's potential negative impact on bone health (44).

A critical consideration in interpreting our findings is the distinction between association and causality. While our data demonstrated significant associations between the use of specific csDMARDs—such as HCQ in RA and MMF in SLE—and an increased prevalence of low BMD, the cross-sectional design of this study precludes definitive causal conclusions. It is highly plausible that the use of these drugs serves as a surrogate marker for higher cumulative disease activity or a more severe disease phenotype. Patients requiring complex immunosuppressive regimens often present with more pronounced systemic inflammation, which is a potent driver of osteoclast activation and bone resorption. Therefore, the observed correlations may reflect the underlying severity of the rheumatic disease rather than direct causative bone-damaging properties.

Specifically, the association between HCQ use and lower BMD in RA and other CTDs may reflect its frequent use in patients with multi-organ involvement or overlap syndromes, who often bear a higher cumulative inflammatory burden. Similarly, MMF is often reserved for more severe, refractory cases of SLE, potentially serving as a surrogate for long-term disease severity and associated bone loss rather than acting as a direct bone-toxic agent.

From a pathophysiological perspective, inflammatory rheumatic diseases and osteoporosis share significant molecular intersections, which are often conceptualised as two sides of the same coin. Chronic systemic inflammation, characterised by elevated levels of pro-inflammatory cytokines including TNF-α, IL-6, and IL-1, has been identified as a significant driver of osteoclastogenesis. These cytokines promote osteoclastogenesis, in part through increased RANKL-mediated signaling, thereby accelerating bone resorption independent of traditional risk factors. Furthermore, in our predominantly female cohort, the postmenopausal decline in oestrogen acts as a crucial synergistic factor; oestrogen deficiency increases skeletal sensitivity to inflammatory cytokines, effectively creating a ‘double-hit’ effect. Coupled with the phenomenon of ‘inflammaging’ (an age-dependent, chronic low-grade inflammatory state), these mechanisms synergistically amplify skeletal damage. Recognising these shared pathways emphasises the importance of clinical management integrating proactive bone health monitoring with anti-inflammatory treatment strategies.

Importantly, a relevant proportion of our low BMD cohort received antiresorptive therapies, specifically bisphosphonates and denosumab. Beyond their primary indication for increasing systemic bone mineral density, these treatments potently inhibit osteoclast-mediated bone resorption. This dual action may have indirect therapeutic implications for the underlying rheumatic condition, particularly by potentially mitigating localized erosive structural joint damage in rheumatoid arthritis. However, assessing structural joint progression over time was beyond the scope of this cross-sectional study.

It is important to note that several limitations must be considered when contextualizing these findings. Regarding our statistical methodology, the significant heterogeneity of disease phenotypes and the complexity of therapeutic regimens necessitated a disease-stratified exploration approach. A global multivariable regression model was considered but ultimately deemed susceptible to overfitting and collinearity, given the limited sample sizes within specific subgroups relative to the high number of clinical covariates.

Firstly, due to the cross-sectional, single-center design at a specialized tertiary rheumatology outpatient clinic, it is not possible to exclude selection bias. As a tertiary referral center, our cohort represents a pre-selected, more severely ill patient population presenting with higher disease complexity, longer disease duration, or accumulated risk factors compared to a general community-based rheumatic population. This accounts for the observed high prevalence of 56% and limits the generalizability of our results to primary care settings.

Secondly, the medication data were restricted to therapies recorded at the time of the study, which precluded the assessment of recently discontinued treatments or long-term therapeutic shifts. Furthermore, certain diagnoses were grouped to maintain statistical power, which may oversimplify complex clinical presentations. For example, the use of hydroxychloroquine in rheumatoid arthritis potentially indicates specific overlap syndromes. Additionally, bone turnover markers, including bone-specific alkaline phosphatase (Ostase) and C-terminal telopeptide (CTX), were not routinely measured, restricting our ability to dynamically evaluate bone metabolism.

Finally, the reliance on self-administered questionnaires introduced potential recall bias or patient uncertainty regarding variables such as bone pain and atraumatic fractures. This insecurity occasionally led to the exclusion of uncertain responses, thereby reducing the database size. For future surveys, it is recommended that questionnaires are reviewed with patients immediately after completion to resolve such uncertainties. The validity of our data was further limited by the availability of exact T-scores from bone density measurements, which were mandatory for standardisation but only available in 352 cases. As a next step to address these challenges, osteoporosis awareness training for practitioners and corresponding evaluations are being planned as part of research projects. Prospective longitudinal studies remain essential to further untangle the complex relationship between medication-specific effects and disease-related inflammatory bone loss.

## 5 Conclusions

Our study demonstrates a substantial and clinically relevant prevalence of low BMD (56%) among patients with inflammatory rheumatic diseases. While we confirmed established clinical risk factors such as advanced age, female gender, and low BMI, our findings highlight a significant association between higher alcohol consumption and low BMD across multiple disease entities, including RA, SLE, and SjS.

Furthermore, the data reveal a strong correlation between the use of specific conventional synthetic DMARDs—particularly HCQ in RA and connective tissue diseases, and MMF in SLE—and increased low BMD prevalence. Although these associations may partly reflect higher underlying disease severity or systemic inflammation, they underscore the necessity for intensified bone health monitoring in these specific treatment groups.

Clinicians should move beyond traditional risk assessments by integrating lifestyle counseling regarding alcohol intake and ensuring early, standardized DXA screenings for patients on complex immunosuppressive regimens. Future longitudinal research is essential to clarify the causal pathways between specific antirheumatic therapies and bone metabolism to ultimately improve fracture prevention in this high-risk population.

## Data Availability

The original contributions presented in the study are included in the article/Supplementary Material, further inquiries can be directed to the corresponding author.

## References

[1] Kanis JA Norton N Harvey NC Jacobson T Johansson H Lorentzon M. SCOPE 2021: a new scorecard for osteoporosis in europe. Arch Osteoporos. (2021) 16:82. 10.1007/s11657-020-00871-9 34080059 PMC8172408

[2] Hadji P Klein S Gothe H Häussler B Kless T Schmidt T. The epidemiology of osteoporosis. Dtsch Ärztebl Int. (2013) 110:52–7. 10.3238/arztebl.2013.0052 PMC3570954 23413388

[3] Johnell O Kanis JA. An estimate of the worldwide prevalence and disability associated with osteoporotic fractures. Osteoporos Int. (2006) 17:1726–33. 10.1007/s00198-006-0172-4 16983459

[4] The REFReSH study group, Leal J Gray AM Prieto-Alhambra D Arden NK Cooper C Javaid MK. Impact of hip fracture on hospital care costs: a population-based study. Osteoporos Int. (2016) 27:549–58. 10.1007/s00198-015-3277-9 26286626 PMC4740562

[5] Jalava T Sarna S Pylkkänen L Mawer B Kanis JA Selby P. Association between vertebral fracture and increased mortality in osteoporotic patients. J Bone Miner Res. (2003) 18:1254–60. 10.1359/jbmr.2003.18.7.1254 12854835

[6] Kanis JA Borgstrom F De Laet C Johansson H Johnell O Jonsson B. Assessment of fracture risk. Osteoporos Int. (2005) 16:581–9. 10.1007/s00198-004-1780-5 15616758

[7] European Prospective Osteoporosis Study (EPOS) Group, Felsenberg D Silman AJ Lunt M Armbrecht G Ismail AA Finn JD. Incidence of vertebral fracture in Europe: results from the European prospective osteoporosis study (EPOS). J Bone Miner Res. (2002) 17:716–24. 10.1359/jbmr.2002.17.4.716 11918229

[8] Siminoski K Leslie WD Frame H Hodsman A Josse RG Khan A. Recommendations for bone mineral density reporting in Canada: a shift to absolute fracture risk assessment. J Clin Densitom. (2007) 10:120–3. 10.1016/j.jocd.2007.01.001 17485028

[9] AWMF Leitlinienregister. Available online at: https://register.awmf.org/de/leitlinien/detail/183-001 (Accessed March 2, 2026)

[10] De Laet C Kanis JA Odén A Johanson H Johnell O Delmas P. Body mass index as a predictor of fracture risk: a meta-analysis. Osteoporos Int. (2005) 16:1330–8. 10.1007/s00198-005-1863-y 15928804

[11] Vosse D Landewé R Van Der Heijde D Van Der Linden S Van Staa T-P Geusens P. Ankylosing spondylitis and the risk of fracture: results from a large primary care-based nested case-control study. Ann Rheum Dis. (2009) 68:1839–42. 10.1136/ard.2008.100503 19066179

[12] Wang X Yan S Liu C Xu Y Wan L Wang Y. Fracture risk and bone mineral density levels in patients with systemic lupus erythematosus: a systematic review and meta-analysis. Osteoporos Int. (2016) 27:1413–23. 10.1007/s00198-015-3449-7 26753541

[13] Liu Y Jiang J Mo M Sun X Yu K. Incidence and risk factors for vertebral fracture in rheumatoid arthritis: an update meta-analysis. Clin Rheumatol. (2022) 41:1313–22. 10.1007/s10067-021-06046-2 35006451

[14] Zink A Braun J Gromnica-Ihle E Krause D Lakomek HJ Mau W. Memorandum der deutschen gesellschaft für rheumatologie zur versorgungsqualität in der rheumatologie—update 2016. Z Für Rheumatol. (2017) 76:195–207. 10.1007/s00393-017-0297-1 28364218

[15] Albrecht K Binder S Minden K Poddubnyy D Regierer AC Strangfeld A. Systematisches review zur schätzung der prävalenz entzündlich rheumatischer erkrankungen in deutschland. Z Für Rheumatol. (2023) 82:727–38. 10.1007/s00393-022-01305-2 PMC10627889 36592211

[16] Adami G Fassio A Rossini M Caimmi C Giollo A Orsolini G. Osteoporosis in rheumatic diseases. Int J Mol Sci. (2019) 20:5867. 10.3390/ijms20235867 31766755 PMC6928928

[17] Mori Y Kuwahara Y Chiba S Kogre A Baba K Kamimura M. Bone mineral density of postmenopausal women with rheumatoid arthritis depends on disease duration regardless of treatment. J Bone Miner Metab. (2017) 35:52–7. 10.1007/s00774-015-0716-9 26369319

[18] Jin S Hsieh E Peng L Yu C Wang Y Wu C. Incidence of fractures among patients with rheumatoid arthritis: a systematic review and meta-analysis. Osteoporos Int. (2018) 29:1263–75. 10.1007/s00198-018-4473-1 29546507

[19] Amin S Gabriel SE Achenbach SJ Atkinson EJ Melton LJ. Are young women and men with rheumatoid arthritis at risk for fragility fractures? A population-based study. J Rheumatol. (2013) 40:1669–76. 10.3899/jrheum.121493 23950189 PMC3910326

[20] Mirza F Canalis E. Management of endocrine disease: secondary osteoporosis: pathophysiology and management. Eur J Endocrinol. (2015) 173:R131–51. 10.1530/EJE-15-0118 25971649 PMC4534332

[21] Pfeil A Lange U. Update DVO-leitlinie 2023 „prophylaxe, diagnostik und therapie der osteoporose bei postmenopausalen frauen und bei männern ab dem 50. lebensjahr“—was ist neu für die rheumatologie? Z Rheumatol. (2024) 83:401–6. 10.1007/s00393-024-01495-x 38512355 PMC11147822

[22] Lee J-H Sung Y-K Choi C-B Cho S-K Bang S-Y Choe J-Y. The frequency of and risk factors for osteoporosis in Korean patients with rheumatoid arthritis. BMC Musculoskelet Disord. (2016) 17:98. 10.1186/s12891-016-0952-8 26912147 PMC4765070

[23] Bultink IEM Lems WF. Lupus and fractures. Curr Opin Rheumatol. (2016) 28:426–32. 10.1097/BOR.0000000000000290 26978130

[24] Smolen JS Landewé RBM Bergstra SA Kerschbaumer A Sepriano A Aletaha D. EULAR recommendations for the management of rheumatoid arthritis with synthetic and biological disease-modifying antirheumatic drugs: 2022 update. Ann Rheum Dis. (2023) 82:3–18. 10.1136/ard-2022-223356 36357155

[25] Abtahi S Cordtz R Dreyer L Driessen JHM Boonen A Burden AM. Biological disease-modifying antirheumatic drugs and osteoporotic fracture risk in patients with rheumatoid arthritis: a Danish cohort study. Am J Med. (2022) 135:879–888.e3. 10.1016/j.amjmed.2022.01.017 35134369

[26] Başkan BM Sivas F Alemdaroğlu E Duran S Özoran K. Association of bone mineral density and vertebral deformity in patients with rheumatoid arthritis. Rheumatol Int. (2007) 27:579–84. 10.1007/s00296-007-0323-8 17287933

[27] Furuya T Kotake S Inoue E Nanke Y Yago T Kobashigawa T. Risk factors associated with incident clinical vertebral and nonvertebral fractures in Japanese women with rheumatoid arthritis: a prospective 54-month observational study. J Rheumatol. (2007) 34:303–10. 17143973

[28] Yan F Wu L Lang J Huang Z. Bone density and fracture risk factors in ankylosing spondylitis: a meta-analysis. Osteoporos Int. (2024) 35:25–40. 10.1007/s00198-023-06925-1 37814094

[29] Ramírez J Nieto-González JC Curbelo Rodríguez R Castañeda S Carmona L. Prevalence and risk factors for osteoporosis and fractures in axial spondyloarthritis: a systematic review and meta-analysis. Semin Arthritis Rheum. (2018) 48:44–52. 10.1016/j.semarthrit.2017.12.001 29290311

[30] Kermani TA Sreih AG Cuthbertson D Carette S Hoffman GS Khalidi NA. Evaluation of damage in giant cell arteritis. Rheumatology. (2018) 57:322–8. 10.1093/rheumatology/kex397 29112740 PMC5850105

[31] Robson J Doll H Suppiah R Flossmann O Harper L Höglund P. Damage in the anca-associated vasculitides: long-term data from the European vasculitis study group (EUVAS) therapeutic trials. Ann Rheum Dis. (2015) 74:177–84. 10.1136/annrheumdis-2013-203927 24243925

[32] Salman-Monte TC Sanchez-Piedra C Fernandez Castro M Andreu JL Martinez Taboada V Olivé A. Prevalence and factors associated with osteoporosis and fragility fractures in patients with primary sjögren syndrome. Rheumatol Int. (2020) 40:1259–65. 10.1007/s00296-020-04615-3 32533289

[33] Azizov V Zaiss MM. Alcohol consumption in rheumatoid arthritis: a path through the immune system. Nutrients. (2021) 13:1324. 10.3390/nu13041324 33923766 PMC8072698

[34] Turk JN Zahavi ER Gorman AE Murray K Turk MA Veale DJ. Exploring the effect of alcohol on disease activity and outcomes in rheumatoid arthritis through systematic review and meta-analysis. Sci Rep. (2021) 11:10474. 10.1038/s41598-021-89618-1 34006854 PMC8131728

[35] Barbhaiya M Lu B Sparks JA Malspeis S Chang S Karlson EW. Influence of alcohol consumption on the risk of systemic lupus erythematosus among women in the Nurses’ health study cohorts. Arthritis Care Res. (2017) 69:384–92. 10.1002/acr.22945 PMC5140763 27273851

[36] Godos J Giampieri F Chisari E Micek A Paladino N Forbes-Hernández TY. Alcohol consumption, bone mineral density, and risk of osteoporotic fractures: a dose–response meta-analysis. Int J Environ Res Public Health. (2022) 19:1515. 10.3390/ijerph19031515 35162537 PMC8835521

[37] Tronstad I Hoff M Horn J Vikjord SAA Videm V Johansson J. Rheumatoid arthritis, disease-modifying antirheumatic drugs and risk of major osteoporotic fracture: prospective data from the HUNT study, Norway. RMD Open. (2024) 10:e003919. 10.1136/rmdopen-2023-003919 38382944 PMC10882308

[38] Carbone L Vasan S Elam R Gupta S Tolaymat O Crandall C. The association of methotrexate, sulfasalazine, and hydroxychloroquine use with fracture in postmenopausal women with rheumatoid arthritis: findings from the women’s health initiative. JBMR Plus. (2020) 4:e10393. 10.1002/jbm4.10393 33103025 PMC7574701

[39] Zerbini CAF Clark P Mendez-Sanchez L Pereira RMR Messina OD Uña CR. Biologic therapies and bone loss in rheumatoid arthritis. Osteoporos Int. (2017) 28:429–46. 10.1007/s00198-016-3769-2 27796445

[40] Hauser B Raterman H Ralston SH Lems WF. The effect of anti-rheumatic drugs on the Skeleton. Calcif Tissue Int. (2022) 111:445–56. 10.1007/s00223-022-01001-y 35771255 PMC9560949

[41] Fürnrohr B Rhodes B Munoz L Weiß K Vyse T Schett G. Osteoclast differentiation is impaired in a subgroup of SLE patients and correlates inversely with mycophenolate mofetil treatment. Int J Mol Sci. (2015) 16:18825–35. 10.3390/ijms160818825 PMC4581274 26274951

[42] Both T Zillikens MC Schreuders-Koedam M Vis M Lam W Weel AEAM. Hydroxychloroquine affects bone resorption both *in vitro* and *in vivo*. J Cell Physiol. (2018) 233:1424–33. 10.1002/jcp.26028 28556961

[43] Panday K Gona A Humphrey MB. Medication-induced osteoporosis: screening and treatment strategies. Ther Adv Musculoskelet Dis. (2014) 6:185–202. 10.1177/1759720X14546350 25342997 PMC4206646

[44] Robin F Cadiou S Albert J-D Bart G Coiffier G Guggenbuhl P. Methotrexate osteopathy: five cases and systematic literature review. Osteoporos Int. (2021) 32:225–32. 10.1007/s00198-020-05664-x 33128074

