# Supplementary material for: Impact of immunosuppressive therapy and clinical risk factors on the prevalence of low bone mineral density in rheumatic diseases
Source: Front Med (Lausanne). 2026 Sep 14;13:1897856. doi: 10.3389/fmed.2026.1897856 (PMC13616625; doi:10.3389/fmed.2026.1897856)
Supplement: Supplementary file 1 [file Datasheet1.docx]

# 1 Supplementary Material 1 English translation of the clinical patient questionnaire

Dear Patient,

Osteoporosis (bone loss) is a disease with far-reaching consequences. If osteoporosis is not diagnosed and treated in time, there is a risk of bone fractures and, as a result, potential pain, surgeries, and limitations in everyday life.

In order to better assess your risk of osteoporosis, we kindly ask you to fill out the following two pages.

1. Have you already been diagnosed with osteoporosis (or a preliminary stage of it)?

☐ Yes ☐ No

2. If YES, have you received any of the medications listed below?

☐ Alendronate (e.g., Fosamax, Binosto, Tevabone, taken once a week)

☐ Ibandronate (e.g., Bonviva, Ascendra, intravenous administration every 3 months)

☐ Risedronate (e.g., Actonel, Acara, taken once a week)

☐ Zoledronate (e.g., Aclasta, Aszora, Zoledon, intravenously once a year)

☐ Teriparatide (e.g., Forsteo, Livogiva, Movymia, Terrosa, subcutaneously once daily for a total of 2 years)

☐ Raloxifene (e.g., Evista, Optruma, taken once daily)

☐ Denosumab (e.g., Prolia every 6 months subcutaneously, Xgeva every 4 weeks subcutaneously)

☐ Romosozumab (e.g., Evenity, 2 subcutaneous injections monthly for a total of 12 months)

☐ other:

3. Are you currently taking medication for osteoporosis?

☐ Yes ☐ No

4. Do you take vitamin D? (e.g., Dekristol, Vigantoletten)

☐ Yes, dose ______ IU daily ☐ No

5. Do you take calcium?

☐ Yes, dose ______ mg daily ☐ No

6. Have you ever had a bone density measurement performed?

☐ Yes, last time in the year _____ ☐ No

7. Are you aware of any bone fractures in your spine?

☐ Yes ☐ No

8. Have bone fractures occurred in other bones without adequate trauma?

☐ Yes	If YES, how often: __________ ☐ No

9. Has your father/mother suffered a femoral neck fracture?

☐ Yes ☐ No

10. Do you fall more frequently than the people around you?

☐ Yes ☐ No

11. Are you physically active for at least 30 minutes every day?

☐ Yes ☐ No

12. What is your diet like?

☐ Omnivore/no restrictions ☐ Vegetarian ☐ Vegan ☐ other: _______________

13. How many cups of coffee_____ / black tea_____ do you drink daily?

14. Do you suffer from any of the following conditions? (Please check all that apply)

☐ Cushing's syndrome and subclinical hypercortisolism

☐ Primary hyperparathyroidism

☐ Growth hormone deficiency due to pituitary insufficiency

☐ Male hypogonadism

☐ Subclinical and manifest hyperthyroidism

☐ Type I diabetes mellitus

☐ Type II diabetes mellitus

☐ Celiac disease and chronic inflammatory bowel diseases

☐ Billroth II gastric resection or gastrectomy

☐ Epilepsy

☐ Depression

☐ Schizophrenia

☐ Stroke (Apoplexy)

☐ Alzheimer's disease

☐ Parkinson's disease

☐ Heart failure

☐ Renal insufficiency

☐ Monoclonal gammopathy of undetermined significance (MGUS)

☐ Genetically confirmed disease, if yes, which one: _________________________

15. Do you take one or more of the following medications? (Please check all that apply)

☐ Tablets for the treatment of prostate cancer

☐ Tablets for the treatment of breast cancer

☐ Tablets for the treatment of epilepsy

☐ Psychotropic drugs

☐ Cortisone

☐ Tablets for the treatment of diabetes (active ingredient glitazone)

☐ Tranquilizers

☐ Sleeping pills

☐ Strong painkillers (opioids/morphines)

☐ Stomach protection (proton pump inhibitors; e.g., omeprazole, pantoprazole)

16. Do you experience bone pain? If yes, how severe is it on a scale from 0 (not at all) to 10 (very severe)?

☐ ☐ ☐ ☐ ☐ ☐ ☐ ☐ ☐ ☐ ☐

0	1	2	3	4	5	6	7	8	9	10

# 2 Supplementary Table 1 Clinical and lifestyle risk factors for low bone mineral density (BMD)

|  | **No Low BMD (T ≤ -1.0)** | | **Low BMD (T ≤ -1.0)** | | **p** |
| --- | --- | --- | --- | --- | --- |
|  | **N** |  | **N** |  |  |
| Gender (N Female, %) | 243/310 | 78.4 | 333/394 | 84.5 | **0.036** |
| Age (years) | 310 | 58.2 ± 12.3 | 394 | 62.9 ± 11.7 | **<0.001** |
| Body Mass Index (kg/m2) | 310 | 28.1 ± 6 | 394 | 25.7 ± 5.6 | **<0.001** |
| Size (cm) | 310 | 169 ± 9.2 | 394 | 166.5 ± 8.3 | **<0.001** |
| Weight (kg) | 310 | 80.3 ± 18.3 | 394 | 71.5 ± 17.3 | **<0.001** |
| Coffee (cups/day) | 310 | 2.1 ± 1.7 | 394 | 2.4 ± 1.6 | 0.066 |
| Tea (cups/day) | 310 | 0.5 ± 1.3 | 394 | 0.5 ± 1.3 | 0.541 |
| Alcohol (glasses/week) | 310 | 0.9 ± 1.7 | 394 | 2.9 ± 1.9 | **<0.001** |
| Cigarettes (N/day) | 310 | 1.7 ± 4.5 | 394 | 10.5 ± 6.6 | **<0.001** |
| Number of sports in a week | 310 | 1.6 ± 1.9 | 394 | 1.7 ± 2.1 | 0.559 |
| 30 min. of exercise daily (N, %) | 265/310 | 85.5 | 326/394 | 82.7 | 0.517 |
| Vertebrae fractures (N, %) | 15/310 | 4.8 | 79/394 | 20.1 | **<0.001** |
| Atraumatic fractures (N, %) | 22/310 | 7.1 | 70/394 | 17.8 | **<0.001** |
| Number of atraumatic fractures | 310 | 1.5 ± 0.7 | 394 | 1.9 ± 1 | 0.085 |
| Parents had femoral neck fracture (N, %) | 29/310 | 9.4 | 48/394 | 12.2 | 0.150 |
| Bone pain (NRS 0-10) | 310 | 3 (0 ~ 6) | 394 | 4 (1 ~ 7) | **0.042** |
| Laboratory values |  | | | | |
| Calcium (mmol/l) | 310 | 2.4 ± 0.1 | 394 | 2.4 ± 0.1 | 0.755 |
| CRP (mg/l) | 310 | 5.1 ± 9.8 | 394 | 3.9 ± 9.7 | 0.102 |
| Serum creatinine (µmol/l) | 310 | 79.5 ± 27.7 | 394 | 81.9 ± 36.1 | 0.317 |
| eGFR (ml/min) | 310 | 79.8 ± 18.2 | 394 | 75.4 ± 19.7 | **0.003** |
| Alkaline phosphatase (U/l) | 310 | 83.2 ± 34.6 | 394 | 76.2 ± 26 | **0.004** |
| Sedimentation rate 1h (mm) | 310 | 13.2 ± 12.9 | 394 | 15.4 ± 13.8 | **0.035** |
| Sedimentation rate 2h (mm) | 310 | 27 ± 19.4 | 394 | 31.2 ± 21.3 | **0.012** |
| Parathyroid hormone (pg/ml) | 310 | 42.5 ± 18.1 | 394 | 48.1 ± 50.6 | **0.048** |
| 25 hydroxyvitamin D (µg/l) | 310 | 31.1 ± 13.2 | 394 | 37 ± 15.1 | **<0.001** |
| Risk factor disease (N, %) | 110/310 | 35.5 | 150/394 | 38.1 | 0.233 |
| Cushing's syndrome (N, %) | 4/310 | 1.3 | 10/394 | 2.5 | 0.207 |
| Primary hyperparathyroidism (N, %) | 0/310 | 0.0 | 2/394 | 0.5 | 0.504 |
| Hyperthyroidism (N, %) | 1/310 | 0.3 | 4/394 | 1.0 | 0.385 |
| Type I diabetes mellitus (N, %) | 7/310 | 2.3 | 6/394 | 1.5 | 0.526 |
| Type II diabetes mellitus (N, %) | 39/310 | 12.6 | 28/394 | 7.1 | **0.024** |
| Celiac disease (N, %) | 10/310 | 3.2 | 19/394 | 4.8 | 0.237 |
| B-II gastric resection/gastrectomy (N, %) | 1/310 | 0.3 | 4/394 | 1.0 | 0.385 |
| Epilepsy (N, %) | 5/310 | 1.6 | 8/394 | 2.0 | 0.623 |
| Depression (N, %) | 30/310 | 9.7 | 37/394 | 9.4 | 0.944 |
| Schizophrenia (N, %) | 1/310 | 0.3 | 0/394 | 0.0 | 0.452 |
| Stroke (N, %) | 6/310 | 1.9 | 7/394 | 1.8 | 0.943 |
| Alzheimer's disease (N, %) | 0/310 | 0.0 | 2/394 | 0.5 | 0.504 |
| Parkinson's disease (N, %) | 0/310 | 0.0 | 1/394 | 0.3 | 1 |
| Cardiac insufficiency (N, %) | 26/310 | 8.4 | 39/394 | 9.9 | 0.377 |
| Renal insufficiency (N, %) | 20/310 | 6.5 | 41/394 | 10.4 | **0.041** |
| MGUS (N, %) | 5/310 | 1.6 | 11/394 | 2.8 | 0.257 |
| Risk factor drug (N, %) | 202/310 | 65.2 | 274/394 | 69.5 | **0.024** |
| Treatment of prostate cancer (N, %) | 0/310 | 0.0 | 2/394 | 0.5 | 0.504 |
| Treatment of breast cancer (N, %) | 4/310 | 1.3 | 2/394 | 0.5 | 0.417 |
| Treatment of epilepsy (N, %) | 4/310 | 1.3 | 9/394 | 2.3 | 0.294 |
| Psychotropic drugs (N, %) | 20/310 | 6.5 | 25/394 | 6.3 | 0.927 |
| Prednisolone (N, %) | 127/310 | 41.0 | 194/394 | 49.2 | **0.006** |
| Glitazone (N, %) | 14/310 | 4.5 | 9/394 | 2.3 | 0.122 |
| Tranquilizers (N, %) | 12/310 | 3.9 | 8/394 | 2.0 | 0.174 |
| Sleeping pills (N, %) | 14/310 | 4.5 | 19/394 | 4.8 | 0.751 |
| Analgesics (N, %) | 26/310 | 8.4 | 57/394 | 14.5 | **0.007** |
| Proton pump inhibitors (N, %) | 118/310 | 38.1 | 174/394 | 44.2 | **0.031** |

**Notes:** Low BMD was defined as described in the Methods. Values are presented as mean ± SD, median [IQR], or n (%) as appropriate.
**Abbreviations:** CRP: C-reactive protein; eGFR: estimated glomerular filtration rate; MGUS: monoclonal gammopathy of undetermined significance; NRS: numeric rating scale.

# 3 Supplementary Table 2 History and current use of specific anti-osteoporotic medications among patients with low bone mineral density (BMD), stratified by rheumatic disease subgroup

| **Diagnosis Group** | **Total Low BMD n*** | **Any History of Treatment n (%)** | **Current Intake n (%)**** | **Alendronate n (%)** | **Ibandronate n (%)** | **Zoledronate  n (%)** | **Denosumab n (%)** |
| --- | --- | --- | --- | --- | --- | --- | --- |
| Total Cohort | 390 | 137 (35.1%) | 76 (20.1%) | 74 (19.0%) | 19 (4.9%) | 9 (2.3%) | 34 (8.7%) |
| Rheumatoid Arthritis | 95 | 39 (41.1%) | 25 (27.8%) | 18 (18.9%) | 6 (6.3%) | 5 (5.3%) | 11 (11.6%) |
| Systemic lupus erythematosus | 68 | 22 (32.4%) | 9 (13.2%) | 16 (23.5%) | 3 (4.4%) | 2 (2.9%) | 4 (5.9%) |
| Spondyloarthritis | 57 | 23 (40.4%) | 13 (23.6%) | 9 (15.8%) | 6 (10.5%) | 1 (1.8%) | 7 (12.3%) |
| Connective tissue disease† | 47 | 13 (27.7%) | 7 (15.2%) | 6 (12.8%) | 1 (2.1%) | 0 (0.0%) | 5 (10.6%) |
| Vasculitis | 46 | 19 (41.3%) | 12 (26.1%) | 13 (28.3%) | 1 (2.2%) | 0 (0.0%) | 3 (6.5%) |
| Sjögren's syndrome | 47 | 13 (27.7%) | 5 (11.6%) | 7 (14.9%) | 1 (2.1%) | 1 (2.1%) | 2 (4.3%) |
| Other | 21 | 6 (28.6%) | 4 (20.0%) | 4 (19.0%) | 1 (4.8%) | 0 (0.0%) | 2 (9.5%) |

**Notes:** Low BMD was defined as described in the Methods. Values are presented as absolute numbers (n) and percentages (%). Subgroups with insufficient valid responses are not shown.
*The total 'n' reflects the number of valid responses regarding medication history within each low BMD subgroup.
**Percentages for 'Current Intake' are calculated based on the available valid data for this specific question, which may differ slightly from the overall subgroup 'n' (e.g., n = 378 for the total cohort).
†Connective tissue diseases excluding Systemic lupus erythematosus and Sjögren's syndrome.
**Abbreviations:** BMD: bone mineral density

# 4 Supplementary Table 3 Significant risk factors and medication associations for low BMD by disease subgroup

| **Diagnosis group** | **Risk factor** | **No Low BMD** | | **Low BMD** | | **p** |
| --- | --- | --- | --- | --- | --- | --- |
|  |  | **N** |  | **N** |  |  |
| Rheumatoid arthritis | Gender (N Female, %) | 33/50 | 66.0 | 83/96 | 86.5 | **0.004** |
|  | Age (years) | 50 | 60.3 ± 11.8 | 96 | 66.7 ± 11.3 | **0.002** |
|  | Body Mass Index (kg/m2) | 50 | 27.6 ± 5.4 | 96 | 25.4 ± 5.7 | **0.025** |
|  | Size (cm) | 50 | 169.5 ± 9.5 | 96 | 165 ± 8.2 | **0.005** |
|  | Weight (kg) | 50 | 78.8 ± 15.3 | 96 | 69.3 ± 16.7 | **0.001** |
|  | Alcohol (glasses/week) | 50 | 1.6 ± 2.8 | 96 | 3.2 ± 1.7 | **0.009** |
|  | Cigarettes (N/day) | 50 | 2.5 ± 4.5 | 96 | 9.5 ± 6.1 | **0.001** |
|  | Vertebrae fractures (N, %) | 1/50 | 2.0 | 25/96 | 26.0 | **<0.001** |
|  | Atraumatic fractures (N, %) | 2/50 | 4.0 | 19/96 | 19.8 | **0.008** |
|  | Number of atraumatic fractures | 50 | 1 ± 0 | 96 | 2.3 ± 1.3 | **0.009** |
|  | CRP (mg/l) | 50 | 10.9 ± 17.8 | 96 | 3.7 ± 8.9 | **0.011** |
|  | Alkaline phosphatase (U/l) | 50 | 91 ± 29.2 | 96 | 76.1 ± 26.1 | **0.005** |
|  | Glitazone (N, %) | 4/50 | 8.0 | 0/96 | 0.0 | **0.016** |
| Systemic lupus erythematosus | Age (years) | 45 | 52.7 ± 11.4 | 68 | 58.8 ± 11.7 | **0.007** |
|  | Body Mass Index (kg/m2) | 45 | 27.9 ± 6.3 | 68 | 25.1 ± 5 | **0.013** |
|  | Weight (kg) | 45 | 80.5 ± 19 | 68 | 69.6 ± 14.7 | **0.002** |
|  | Alcohol (glasses/week) | 45 | 1 ± 1.5 | 68 | 2.6 ± 1 | **<0.001** |
|  | Cigarettes (N/day) | 45 | 2.5 ± 4.5 | 68 | 9.6 ± 7.1 | **0.039** |
| Spondyloarthritis | Body Mass Index (kg/m2) | 68 | 30 ± 5.7 | 58 | 26.4 ± 5.7 | **0.001** |
|  | Weight (kg) | 68 | 86.2 ± 17.1 | 58 | 75.4 ± 18.6 | **0.001** |
|  | Cigarettes (N/day) | 68 | 0.8 ± 2.3 | 58 | 12.5 ± 8 | **0.001** |
|  | Vertebrae fractures (N, %) | 2/68 | 2.9 | 10/58 | 17.2 | **0.005** |
|  | Atraumatic fractures (N, %) | 7/68 | 10.3 | 16/58 | 27.6 | **0.008** |
|  | Type II diabetes mellitus (N, %) | 13/68 | 19.1 | 4/58 | 6.9 | **0.033** |
| Connective tissue disease* | Body Mass Index (kg/m2) | 35 | 27.5 ± 5.6 | 47 | 24.5 ± 4.9 | **0.014** |
|  | Weight (kg) | 35 | 78.1 ± 18.1 | 47 | 67.8 ± 15.8 | **0.009** |
|  | Tea (cups/day) | 35 | 0.6 ± 1 | 47 | 0.1 ± 0.5 | **0.026** |
|  | Alcohol (glasses/week) | 35 | 0.8 ± 1.3 | 47 | 3.4 ± 2.1 | **0.015** |
|  | Cigarettes (N/day) | 35 | 0 ± 0 | 47 | 10.6 ± 5.9 | **0.003** |
|  | Vertebrae fractures (N, %) | 0/35 | 0.0 | 11/47 | 23.4 | **0.002** |
|  | Cardiac insufficiency (N, %) | 4/35 | 11.4 | 0/47 | 0.0 | **0.033** |
|  | Analgesics (N, %) | 0/35 | 0.0 | 7/47 | 14.9 | **0.017** |
| Vasculitis | eGFR (ml/min) | 32 | 79.1 ± 24.7 | 47 | 68.1 ± 21.4 | **0.049** |
|  | Cigarettes (N/day) | 32 | 3.3 ± 6.1 | 47 | 13.9 ± 5 | **0.015** |
|  | Sedimentation rate 1h (mm) | 32 | 9.8 ± 6 | 47 | 14.7 ± 13.6 | **0.046** |
| Sjögren's syndrome | Alcohol (glasses/week) | 32 | 0.2 ± 0.7 | 47 | 3.2 ± 2.3 | **0.014** |
|  | Vertebrae fractures (N, %) | 2/32 | 6.3 | 13/47 | 27.7 | **0.013** |
|  | Alkaline phosphatase (U/l) | 32 | 88.7 ± 39.6 | 47 | 71.9 ± 21.1 | **0.039** |
| Other | Bone pain (NRS 0-10) | 30 | 0.5 (0 ~ 5.25) | 21 | 5 (4.5 ~ 7.5) | **0.034** |
|  | Parents had femoral neck fracture (N, %) | 1/30 | 3.3 | 5/21 | 23.8 | **0.022** |
| Autoinflammation | Age (years) | 6 | 41.2 ± 10.2 | 7 | 61.1 ± 16.8 | **0.025** |
| Undiagnosed | eGFR (ml/min) | 12 | 79.3 ± 20.9 | 3 | 62.7 ± 1.5 | **0.019** |
|  | Vertebrae fractures (N, %) | 1/12 | 8.3 | 3/3 | 100.0 | **0.009** |

**Notes:** Low BMD was defined as described in the Methods. Values are presented as mean ± SD or n/N (%).
*Connective tissue diseases excluding SLE and SjS.
**Abbreviations:** CRP: C-reactive protein; eGFR: estimated glomerular filtration rate; NRS: numeric rating scale.

# 5 Supplementary Table 4 Comprehensive analysis of osteoporosis risk factors across all diagnostic subgroups

| **Diagnosis group** | **Risk factor** | **No Low BMD** | | **Low BMD** | | **p** |
| --- | --- | --- | --- | --- | --- | --- |
|  |  | **N** |  | **N** |  |  |
| **Rheumatoid arthritis** | Gender (N Female, %) | 33/50 | 66.0 | 83/96 | 86.5 | **0.004** |
|  | Age (years) | 50 | 60.3 ± 11.8 | 96 | 66.7 ± 11.3 | **0.002** |
|  | Body Mass Index (kg/m2) | 50 | 27.6 ± 5.4 | 96 | 25.4 ± 5.7 | **0.025** |
|  | Size (cm) | 50 | 169.5 ± 9.5 | 96 | 165.0 ± 8.2 | **0.005** |
|  | Weight (kg) | 50 | 78.8 ± 15.3 | 96 | 69.3 ± 16.7 | **0.001** |
|  | Coffee (cups/day) | 50 | 1.8 ± 1.3 | 96 | 2.3 ± 1.7 | 0.076 |
|  | Tea (cups/day) | 50 | 0.5 ± 1.3 | 96 | 0.5 ± 1.2 | 0.846 |
|  | Alcohol (glasses/week) | 50 | 1.6 ± 2.8 | 96 | 3.2 ± 1.7 | **0.009** |
|  | Cigarettes (N/day) | 50 | 2.5 ± 4.5 | 96 | 9.5 ± 6.1 | **0.001** |
|  | Number of sports in a week | 50 | 1.4 ± 1.8 | 96 | 2.0 ± 2.2 | 0.132 |
|  | 30 min. of exercise daily (N, %) | 44/50 | 88.0 | 84/96 | 87.5 | 0.326 |
|  | Vertebrae fractures (N, %) | 1/50 | 2.0 | 25/96 | 26.0 | **<0.001** |
|  | Atraumatic fractures (N, %) | 2/50 | 4.0 | 19/96 | 19.8 | **0.008** |
|  | Number of atraumatic fractures | 50 | 1.0 ± 0.0 | 96 | 2.3 ± 1.3 | **0.009** |
|  | Parents had femoral neck fracture (N, %) | 8/50 | 16.0 | 11/96 | 11.5 | 0.501 |
|  | Bone pain (NRS 0-10) | 50 | 2 (0 ~ 6) | 96 | 4 (1 ~ 6) | 0.154 |
|  | Laboratory values |  | | | | |
|  | Calcium (mmol/l) | 50 | 2.3 ± 0.1 | 96 | 2.4 ± 0.1 | 0.607 |
|  | CRP (mg/l) | 50 | 10.9 ± 17.8 | 96 | 3.7 ± 8.9 | **0.011** |
|  | Serum creatinine (µmol/l) | 50 | 82.0 ± 28.0 | 96 | 78.9 ± 21.9 | 0.508 |
|  | eGFR (ml/min) | 50 | 78.8 ± 20.3 | 96 | 73.6 ± 17.6 | 0.144 |
|  | Alkaline phosphatase (U/l) | 50 | 91.0 ± 29.2 | 96 | 76.1 ± 26.1 | **0.005** |
|  | Sedimentation rate 1h (mm) | 50 | 15.7 ± 16.3 | 96 | 12.8 ± 9.8 | 0.282 |
|  | Sedimentation rate 2h (mm) | 50 | 30.9 ± 23.5 | 96 | 27.6 ± 19.9 | 0.428 |
|  | Parathyroid hormone (pg/ml) | 50 | 41.7 ± 23.9 | 96 | 50.3 ± 41.9 | 0.132 |
|  | 25 hydroxyvitamin D (µg/l) | 50 | 28.3 ± 14.2 | 96 | 37.9 ± 17.3 | **0.014** |
|  | Risk factor disease (N, %) | 19/50 | 38.0 | 30/96 | 31.3 | 0.611 |
|  | Cushing's syndrome (N, %) | 0/50 | 0.0 | 2/96 | 2.1 | 0.536 |
|  | Primary hyperparathyroidism (N, %) | 0/50 | 0.0 | 0/96 | 0.0 | - |
|  | Growth hormone deficiency (N, %) | 0/50 | 0.0 | 0/96 | 0.0 | - |
|  | Hyperthyroidism (N, %) | 0/50 | 0.0 | 0/96 | 0.0 | - |
|  | Type I diabetes mellitus (N, %) | 1/50 | 2.0 | 3/96 | 3.1 | >0.999 |
|  | Type II diabetes mellitus (N, %) | 8/50 | 16.0 | 6/96 | 6.3 | 0.082 |
|  | Celiac disease (N, %) | 1/50 | 2.0 | 1/96 | 1.0 | >0.999 |
|  | B-II gastric resection/gastrectomy (N, %) | 0/50 | 0.0 | 1/96 | 1.0 | >0.999 |
|  | Epilepsy (N, %) | 2/50 | 4.0 | 0/96 | 0.0 | 0.128 |
|  | Depression (N, %) | 4/50 | 8.0 | 8/96 | 8.3 | >0.999 |
|  | Schizophrenia (N, %) | 0/50 | 0.0 | 0/96 | 0.0 | - |
|  | Stroke (N, %) | 1/50 | 2.0 | 0/96 | 0.0 | 0.360 |
|  | Alzheimer's disease (N, %) | 0/50 | 0.0 | 0/96 | 0.0 | - |
|  | Parkinson's disease (N, %) | 0/50 | 0.0 | 1/96 | 1.0 | >0.999 |
|  | Cardiac insufficiency (N, %) | 3/50 | 6.0 | 10/96 | 10.4 | 0.377 |
|  | Renal insufficiency (N, %) | 2/50 | 4.0 | 10/96 | 10.4 | 0.211 |
|  | MGUS (N, %) | 1/50 | 2.0 | 0/96 | 0.0 | 0.360 |
|  | Risk factor drug (N, %) | 33/50 | 66.0 | 58/96 | 60.4 | 0.991 |
|  | Treatment of prostate cancer (N, %) | 0/50 | 0.0 | 0/96 | 0.0 | - |
|  | Treatment of breast cancer (N, %) | 1/50 | 2.0 | 1/96 | 1.0 | >0.999 |
|  | Treatment of epilepsy (N, %) | 1/50 | 2.0 | 0/96 | 0.0 | 0.362 |
|  | Psychotropic drugs (N, %) | 4/50 | 8.0 | 1/96 | 1.0 | 0.058 |
|  | Prednisolone (N, %) | 21/50 | 42.0 | 45/96 | 46.9 | 0.302 |
|  | Glitazone (N, %) | 4/50 | 8.0 | 0/96 | 0.0 | **0.016** |
|  | Tranquilizers (N, %) | 2/50 | 4.0 | 2/96 | 2.1 | 0.621 |
|  | Sleeping pills (N, %) | 2/50 | 4.0 | 4/96 | 4.2 | >0.999 |
|  | Analgesics (N, %) | 2/50 | 4.0 | 13/96 | 13.5 | 0.051 |
|  | Proton pump inhibitors (N, %) | 16/50 | 32.0 | 32/96 | 33.3 | 0.605 |
| **Systemic lupus erythematosus** | Gender (N Female, %) | 39/45 | 86.7 | 61/68 | 89.7 | 0.620 |
|  | Age (years) | 45 | 52.7 ± 11.4 | 68 | 58.8 ± 11.7 | **0.007** |
|  | Body Mass Index (kg/m2) | 45 | 27.9 ± 6.3 | 68 | 25.1 ± 5.0 | **0.013** |
|  | Size (cm) | 45 | 169.5 ± 8.5 | 68 | 166.5 ± 6.4 | 0.051 |
|  | Weight (kg) | 45 | 80.5 ± 19.0 | 68 | 69.6 ± 14.7 | **0.002** |
|  | Coffee (cups/day) | 45 | 2.2 ± 1.8 | 68 | 2.5 ± 1.5 | 0.432 |
|  | Tea (cups/day) | 45 | 0.5 ± 0.9 | 68 | 0.3 ± 0.7 | 0.195 |
|  | Alcohol (glasses/week) | 45 | 1.0 ± 1.5 | 68 | 2.6 ± 1.0 | **<0.001** |
|  | Cigarettes (N/day) | 45 | 2.5 ± 4.5 | 68 | 9.6 ± 7.1 | **0.039** |
|  | Number of sports in a week | 45 | 1.4 ± 1.5 | 68 | 1.9 ± 2.5 | 0.146 |
|  | 30 min. of exercise daily (N, %) | 40/45 | 88.9 | 56/68 | 82.4 | >0.999 |
|  | Vertebrae fractures (N, %) | 5/45 | 11.1 | 11/68 | 16.2 | 0.413 |
|  | Atraumatic fractures (N, %) | 2/45 | 4.4 | 9/68 | 13.2 | 0.194 |
|  | Number of atraumatic fractures | 45 | 1.5 ± 0.7 | 68 | 1.6 ± 1.0 | 0.918 |
|  | Parents had femoral neck fracture (N, %) | 4/45 | 8.9 | 3/68 | 4.4 | 0.438 |
|  | Bone pain (NRS 0-10) | 45 | 3 (0 ~ 6) | 68 | 2 (0 ~ 6) | 0.395 |
|  | Laboratory values |  | | | | |
|  | Calcium (mmol/l) | 45 | 2.3 ± 0.1 | 68 | 2.3 ± 0.1 | 0.933 |
|  | CRP (mg/l) | 45 | 2.9 ± 4.0 | 68 | 2.8 ± 4.0 | 0.937 |
|  | Serum creatinine (µmol/l) | 45 | 75.3 ± 14.1 | 68 | 78.6 ± 32.3 | 0.463 |
|  | eGFR (ml/min) | 45 | 83.0 ± 14.4 | 68 | 79.6 ± 19.4 | 0.292 |
|  | Alkaline phosphatase (U/l) | 45 | 80.8 ± 31.8 | 68 | 75.2 ± 29.8 | 0.361 |
|  | Sedimentation rate 1h (mm) | 45 | 12.4 ± 10.5 | 68 | 16.7 ± 12.9 | 0.063 |
|  | Sedimentation rate 2h (mm) | 45 | 26.6 ± 20.6 | 68 | 34.2 ± 20.6 | 0.067 |
|  | Parathyroid hormone (pg/ml) | 45 | 40.4 ± 12.6 | 68 | 40.7 ± 19.5 | 0.939 |
|  | 25 hydroxyvitamin D (µg/l) | 45 | 35.1 ± 14.1 | 68 | 35.4 ± 12.1 | 0.923 |
|  | Risk factor disease (N, %) | 11/45 | 24.4 | 29/68 | 42.6 | **0.028** |
|  | Cushing's syndrome (N, %) | 0/45 | 0.0 | 1/68 | 1.5 | >0.999 |
|  | Primary hyperparathyroidism (N, %) | 0/45 | 0.0 | 1/68 | 1.5 | >0.999 |
|  | Growth hormone deficiency (N, %) | 0/45 | 0.0 | 0/68 | 0.0 | - |
|  | Hyperthyroidism (N, %) | 1/45 | 2.2 | 2/68 | 2.9 | >0.999 |
|  | Type I diabetes mellitus (N, %) | 0/45 | 0.0 | 1/68 | 1.5 | >0.999 |
|  | Type II diabetes mellitus (N, %) | 2/45 | 4.4 | 3/68 | 4.4 | >0.999 |
|  | Celiac disease (N, %) | 0/45 | 0.0 | 5/68 | 7.4 | 0.077 |
|  | B-II gastric resection/gastrectomy (N, %) | 0/45 | 0.0 | 0/68 | 0.0 | - |
|  | Epilepsy (N, %) | 0/45 | 0.0 | 0/68 | 0.0 | - |
|  | Depression (N, %) | 6/45 | 13.3 | 7/68 | 10.3 | 0.684 |
|  | Schizophrenia (N, %) | 0/45 | 0.0 | 0/68 | 0.0 | - |
|  | Stroke (N, %) | 2/45 | 4.4 | 4/68 | 5.9 | >0.999 |
|  | Alzheimer's disease (N, %) | 0/45 | 0.0 | 2/68 | 2.9 | 0.512 |
|  | Parkinson's disease (N, %) | 0/45 | 0.0 | 0/68 | 0.0 | - |
|  | Cardiac insufficiency (N, %) | 1/45 | 2.2 | 8/68 | 11.8 | 0.079 |
|  | Renal insufficiency (N, %) | 1/45 | 2.2 | 8/68 | 11.8 | 0.079 |
|  | MGUS (N, %) | 0/45 | 0.0 | 4/68 | 5.9 | 0.143 |
|  | Risk factor drug (N, %) | 28/45 | 62.2 | 55/68 | 80.9 | **0.007** |
|  | Treatment of prostate cancer (N, %) | 0/45 | 0.0 | 1/68 | 1.5 | >0.999 |
|  | Treatment of breast cancer (N, %) | 1/45 | 2.2 | 1/68 | 1.5 | >0.999 |
|  | Treatment of epilepsy (N, %) | 1/45 | 2.2 | 1/68 | 1.5 | >0.999 |
|  | Psychotropic drugs (N, %) | 4/45 | 8.9 | 7/68 | 10.3 | >0.999 |
|  | Prednisolone (N, %) | 21/45 | 46.7 | 41/68 | 60.3 | 0.096 |
|  | Glitazone (N, %) | 0/45 | 0.0 | 3/68 | 4.4 | 0.270 |
|  | Tranquilizers (N, %) | 1/45 | 2.2 | 2/68 | 2.9 | >0.999 |
|  | Sleeping pills (N, %) | 0/45 | 0.0 | 3/68 | 4.4 | 0.270 |
|  | Analgesics (N, %) | 4/45 | 8.9 | 7/68 | 10.3 | >0.999 |
|  | Proton pump inhibitors (N, %) | 16/45 | 35.6 | 31/68 | 45.6 | 0.222 |
| **Spondyloarthritis** | Gender (N Female, %) | 52/68 | 76.5 | 47/58 | 81.0 | 0.534 |
|  | Age (years) | 68 | 58.8 ± 9.8 | 58 | 59.3 ± 11.6 | 0.788 |
|  | Body Mass Index (kg/m2) | 68 | 30.0 ± 5.7 | 58 | 26.4 ± 5.7 | **0.001** |
|  | Size (cm) | 68 | 169.6 ± 9.0 | 58 | 168.8 ± 8.2 | 0.577 |
|  | Weight (kg) | 68 | 86.2 ± 17.1 | 58 | 75.4 ± 18.6 | **0.001** |
|  | Coffee (cups/day) | 68 | 2.4 ± 1.7 | 58 | 2.8 ± 1.8 | 0.234 |
|  | Tea (cups/day) | 68 | 0.5 ± 1.1 | 58 | 0.5 ± 1.4 | 0.865 |
|  | Alcohol (glasses/week) | 68 | 1.0 ± 1.5 | 58 | 2.1 ± 1.6 | 0.059 |
|  | Cigarettes (N/day) | 68 | 0.8 ± 2.3 | 58 | 12.5 ± 8.0 | **0.001** |
|  | Number of sports in a week | 68 | 1.9 ± 1.9 | 58 | 1.9 ± 2.0 | 0.876 |
|  | 30 min. of exercise daily (N, %) | 61/68 | 89.7 | 52/58 | 89.7 | 0.255 |
|  | Vertebrae fractures (N, %) | 2/68 | 2.9 | 10/58 | 17.2 | **0.005** |
|  | Atraumatic fractures (N, %) | 7/68 | 10.3 | 16/58 | 27.6 | **0.008** |
|  | Number of atraumatic fractures | 68 | 1.5 ± 0.8 | 58 | 1.9 ± 0.7 | 0.335 |
|  | Parents had femoral neck fracture (N, %) | 6/68 | 8.8 | 9/58 | 15.5 | 0.201 |
|  | Bone pain (NRS 0-10) | 68 | 4.5 (1.25 ~ 7) | 58 | 6 (4 ~ 7) | 0.190 |
|  | Laboratory values |  | | | | |
|  | Calcium (mmol/l) | 68 | 2.4 ± 0.1 | 58 | 2.4 ± 0.1 | 0.689 |
|  | CRP (mg/l) | 68 | 4.4 ± 5.0 | 58 | 4.2 ± 9.3 | 0.880 |
|  | Serum creatinine (µmol/l) | 68 | 80.6 ± 39.8 | 58 | 75.7 ± 19.0 | 0.383 |
|  | eGFR (ml/min) | 68 | 80.0 ± 16.8 | 58 | 81.3 ± 17.3 | 0.681 |
|  | Alkaline phosphatase (U/l) | 68 | 79.4 ± 24.2 | 58 | 80.2 ± 25.0 | 0.874 |
|  | Sedimentation rate 1h (mm) | 68 | 11.5 ± 7.5 | 58 | 14.0 ± 9.3 | 0.120 |
|  | Sedimentation rate 2h (mm) | 68 | 24.7 ± 16.0 | 58 | 30.7 ± 19.1 | 0.079 |
|  | Parathyroid hormone (pg/ml) | 68 | 42.9 ± 15.2 | 58 | 41.7 ± 17.3 | 0.696 |
|  | 25 hydroxyvitamin D (µg/l) | 68 | 26.9 ± 11.2 | 58 | 35.1 ± 13.4 | **0.014** |
|  | Risk factor disease (N, %) | 29/68 | 42.6 | 23/58 | 39.7 | 0.531 |
|  | Cushing's syndrome (N, %) | 1/68 | 1.5 | 2/58 | 3.4 | 0.604 |
|  | Primary hyperparathyroidism (N, %) | 0/68 | 0.0 | 1/58 | 1.7 | 0.475 |
|  | Growth hormone deficiency (N, %) | 0/68 | 0.0 | 0/58 | 0.0 | - |
|  | Hyperthyroidism (N, %) | 0/68 | 0.0 | 0/58 | 0.0 | - |
|  | Type I diabetes mellitus (N, %) | 3/68 | 4.4 | 0/58 | 0.0 | 0.246 |
|  | Type II diabetes mellitus (N, %) | 13/68 | 19.1 | 4/58 | 6.9 | **0.033** |
|  | Celiac disease (N, %) | 7/68 | 10.3 | 9/58 | 15.5 | 0.452 |
|  | B-II gastric resection/gastrectomy (N, %) | 1/68 | 1.5 | 0/58 | 0.0 | >0.999 |
|  | Epilepsy (N, %) | 2/68 | 2.9 | 3/58 | 5.2 | 0.667 |
|  | Depression (N, %) | 5/68 | 7.4 | 7/58 | 12.1 | 0.428 |
|  | Schizophrenia (N, %) | 1/68 | 1.5 | 0/58 | 0.0 | >0.999 |
|  | Stroke (N, %) | 2/68 | 2.9 | 0/58 | 0.0 | 0.497 |
|  | Alzheimer's disease (N, %) | 0/68 | 0.0 | 0/58 | 0.0 | - |
|  | Parkinson's disease (N, %) | 0/68 | 0.0 | 0/58 | 0.0 | - |
|  | Cardiac insufficiency (N, %) | 7/68 | 10.3 | 3/58 | 5.2 | 0.329 |
|  | Renal insufficiency (N, %) | 7/68 | 10.3 | 4/58 | 6.9 | 0.438 |
|  | MGUS (N, %) | 2/68 | 2.9 | 1/58 | 1.7 | >0.999 |
|  | Risk factor drug (N, %) | 43/68 | 63.2 | 40/58 | 69.0 | 0.820 |
|  | Treatment of prostate cancer (N, %) | 0/68 | 0.0 | 0/58 | 0.0 | - |
|  | Treatment of breast cancer (N, %) | 0/68 | 0.0 | 0/58 | 0.0 | - |
|  | Treatment of epilepsy (N, %) | 0/68 | 0.0 | 3/58 | 5.2 | 0.104 |
|  | Psychotropic drugs (N, %) | 3/68 | 4.4 | 4/58 | 6.9 | 0.707 |
|  | Prednisolone (N, %) | 18/68 | 26.5 | 23/58 | 39.7 | 0.174 |
|  | Glitazone (N, %) | 3/68 | 4.4 | 1/58 | 1.7 | 0.621 |
|  | Tranquilizers (N, %) | 2/68 | 2.9 | 1/58 | 1.7 | >0.999 |
|  | Sleeping pills (N, %) | 2/68 | 2.9 | 4/58 | 6.9 | 0.422 |
|  | Analgesics (N, %) | 12/68 | 17.6 | 18/58 | 31.0 | 0.113 |
|  | Proton pump inhibitors (N, %) | 35/68 | 51.5 | 32/58 | 55.2 | 0.949 |
| **Connective tissue disease*** | Gender (N Female, %) | 29/35 | 82.9 | 39/47 | 83.0 | 0.988 |
|  | Age (years) | 35 | 57.3 ± 13.2 | 47 | 61.1 ± 12.4 | 0.193 |
|  | Body Mass Index (kg/m2) | 35 | 27.5 ± 5.6 | 47 | 24.5 ± 4.9 | **0.014** |
|  | Size (cm) | 35 | 168.3 ± 8.7 | 47 | 165.8 ± 8.4 | 0.197 |
|  | Weight (kg) | 35 | 78.1 ± 18.1 | 47 | 67.8 ± 15.8 | **0.009** |
|  | Coffee (cups/day) | 35 | 2.3 ± 1.6 | 47 | 2.0 ± 1.7 | 0.425 |
|  | Tea (cups/day) | 35 | 0.6 ± 1.0 | 47 | 0.1 ± 0.5 | **0.026** |
|  | Alcohol (glasses/week) | 35 | 0.8 ± 1.3 | 47 | 3.4 ± 2.1 | **0.015** |
|  | Cigarettes (N/day) | 35 | 0.0 ± 0.0 | 47 | 10.6 ± 5.9 | **0.003** |
|  | Number of sports in a week | 35 | 2.1 ± 2.2 | 47 | 1.2 ± 1.7 | 0.068 |
|  | 30 min. of exercise daily (N, %) | 31/35 | 88.6 | 38/47 | 80.9 | 0.231 |
|  | Vertebrae fractures (N, %) | 0/35 | 0.0 | 11/47 | 23.4 | **0.002** |
|  | Atraumatic fractures (N, %) | 4/35 | 11.4 | 4/47 | 8.5 | >0.999 |
|  | Number of atraumatic fractures | 35 | 1.2 ± 0.5 | 47 | 2.0 ± 1.2 | 0.298 |
|  | Parents had femoral neck fracture (N, %) | 4/35 | 11.4 | 5/47 | 10.6 | >0.999 |
|  | Bone pain (NRS 0-10) | 35 | 2.5 (0 ~ 6.5) | 47 | 4 (0 ~ 6.5) | 0.696 |
|  | Laboratory values |  | | | | |
|  | Calcium (mmol/l) | 35 | 2.4 ± 0.1 | 47 | 2.4 ± 0.1 | 0.190 |
|  | CRP (mg/l) | 35 | 3.9 ± 8.7 | 47 | 6.4 ± 21.2 | 0.486 |
|  | Serum creatinine (µmol/l) | 35 | 79.5 ± 22.1 | 47 | 77.9 ± 21.8 | 0.740 |
|  | eGFR (ml/min) | 35 | 79.5 ± 18.6 | 47 | 78.7 ± 21.5 | 0.867 |
|  | Alkaline phosphatase (U/l) | 35 | 78.4 ± 30.9 | 47 | 74.5 ± 25.2 | 0.544 |
|  | Sedimentation rate 1h (mm) | 35 | 12.3 ± 10.3 | 47 | 17.3 ± 12.9 | 0.069 |
|  | Sedimentation rate 2h (mm) | 35 | 26.2 ± 18.5 | 47 | 34.4 ± 20.4 | 0.076 |
|  | Parathyroid hormone (pg/ml) | 35 | 47.4 ± 21.8 | 47 | 44.8 ± 23.7 | 0.625 |
|  | 25 hydroxyvitamin D (µg/l) | 35 | 29.3 ± 8.7 | 47 | 40.0 ± 18.9 | 0.050 |
|  | Risk factor disease (N, %) | 11/35 | 31.4 | 19/47 | 40.4 | 0.330 |
|  | Cushing's syndrome (N, %) | 1/35 | 2.9 | 2/47 | 4.3 | >0.999 |
|  | Primary hyperparathyroidism (N, %) | 0/35 | 0.0 | 0/47 | 0.0 | - |
|  | Growth hormone deficiency (N, %) | 0/35 | 0.0 | 0/47 | 0.0 | - |
|  | Hyperthyroidism (N, %) | 0/35 | 0.0 | 0/47 | 0.0 | - |
|  | Type I diabetes mellitus (N, %) | 1/35 | 2.9 | 1/47 | 2.1 | >0.999 |
|  | Type II diabetes mellitus (N, %) | 3/35 | 8.6 | 2/47 | 4.3 | 0.648 |
|  | Celiac disease (N, %) | 0/35 | 0.0 | 2/47 | 4.3 | 0.502 |
|  | B-II gastric resection/gastrectomy (N, %) | 0/35 | 0.0 | 2/47 | 4.3 | 0.502 |
|  | Epilepsy (N, %) | 0/35 | 0.0 | 0/47 | 0.0 | - |
|  | Depression (N, %) | 3/35 | 8.6 | 5/47 | 10.6 | >0.999 |
|  | Schizophrenia (N, %) | 0/35 | 0.0 | 0/47 | 0.0 | - |
|  | Stroke (N, %) | 0/35 | 0.0 | 0/47 | 0.0 | - |
|  | Alzheimer's disease (N, %) | 0/35 | 0.0 | 0/47 | 0.0 | - |
|  | Parkinson's disease (N, %) | 0/35 | 0.0 | 0/47 | 0.0 | - |
|  | Cardiac insufficiency (N, %) | 4/35 | 11.4 | 0/47 | 0.0 | **0.033** |
|  | Renal insufficiency (N, %) | 5/35 | 14.3 | 2/47 | 4.3 | 0.229 |
|  | MGUS (N, %) | 1/35 | 2.9 | 2/47 | 4.3 | >0.999 |
|  | Risk factor drug (N, %) | 23/35 | 65.7 | 37/47 | 78.7 | 0.087 |
|  | Treatment of prostate cancer (N, %) | 0/35 | 0.0 | 0/47 | 0.0 | - |
|  | Treatment of breast cancer (N, %) | 1/35 | 2.9 | 0/47 | 0.0 | 0.436 |
|  | Treatment of epilepsy (N, %) | 0/35 | 0.0 | 1/47 | 2.1 | >0.999 |
|  | Psychotropic drugs (N, %) | 2/35 | 5.7 | 3/47 | 6.4 | >0.999 |
|  | Prednisolone (N, %) | 14/35 | 40.0 | 23/47 | 48.9 | 0.330 |
|  | Glitazone (N, %) | 1/35 | 2.9 | 2/47 | 4.3 | >0.999 |
|  | Tranquilizers (N, %) | 0/35 | 0.0 | 1/47 | 2.1 | >0.999 |
|  | Sleeping pills (N, %) | 0/35 | 0.0 | 3/47 | 6.4 | 0.253 |
|  | Analgesics (N, %) | 0/35 | 0.0 | 7/47 | 14.9 | **0.017** |
|  | Proton pump inhibitors (N, %) | 16/35 | 45.7 | 25/47 | 53.2 | 0.392 |
| **Vasculitis** | Gender (N Female, %) | 25/32 | 78.1 | 36/47 | 76.6 | 0.874 |
|  | Age (years) | 32 | 58.6 ± 15.8 | 47 | 64.3 ± 10.2 | 0.076 |
|  | Body Mass Index (kg/m2) | 32 | 28.2 ± 7.3 | 47 | 27.1 ± 6.7 | 0.515 |
|  | Size (cm) | 32 | 167.2 ± 8.4 | 47 | 168.1 ± 9.3 | 0.671 |
|  | Weight (kg) | 32 | 78.5 ± 19.0 | 47 | 76.7 ± 19.6 | 0.683 |
|  | Coffee (cups/day) | 32 | 2.0 ± 1.3 | 47 | 2.4 ± 1.5 | 0.212 |
|  | Tea (cups/day) | 32 | 0.5 ± 1.3 | 47 | 0.6 ± 2.0 | 0.878 |
|  | Alcohol (glasses/week) | 32 | 0.6 ± 1.5 | 47 | 4.8 ± 4.4 | 0.102 |
|  | Cigarettes (N/day) | 32 | 3.3 ± 6.1 | 47 | 13.9 ± 5.0 | **0.015** |
|  | Number of sports in a week | 32 | 1.2 ± 1.8 | 47 | 1.5 ± 1.9 | 0.572 |
|  | 30 min. of exercise daily (N, %) | 26/32 | 81.3 | 39/47 | 83.0 | 0.132 |
|  | Vertebrae fractures (N, %) | 2/32 | 6.3 | 4/47 | 8.5 | >0.999 |
|  | Atraumatic fractures (N, %) | 1/32 | 3.1 | 9/47 | 19.1 | 0.078 |
|  | Number of atraumatic fractures | 32 | 1.0 | 47 | 1.7 ± 1.0 | - |
|  | Parents had femoral neck fracture (N, %) | 1/32 | 3.1 | 4/47 | 8.5 | 0.643 |
|  | Bone pain (NRS 0-10) | 32 | 2 (0 ~ 6) | 47 | 3 (0 ~ 5.25) | 0.950 |
|  | Laboratory values |  | | | | |
|  | Calcium (mmol/l) | 32 | 2.4 ± 0.1 | 47 | 2.4 ± 0.1 | 0.883 |
|  | CRP (mg/l) | 32 | 3.7 ± 4.9 | 47 | 3.9 ± 3.7 | 0.871 |
|  | Serum creatinine (µmol/l) | 32 | 83.2 ± 33.1 | 47 | 97.6 ± 66.4 | 0.218 |
|  | eGFR (ml/min) | 32 | 79.1 ± 24.7 | 47 | 68.1 ± 21.4 | **0.049** |
|  | Alkaline phosphatase (U/l) | 32 | 80.1 ± 23.6 | 47 | 78.5 ± 26.6 | 0.788 |
|  | Sedimentation rate 1h (mm) | 32 | 9.8 ± 6.0 | 47 | 14.7 ± 13.6 | **0.046** |
|  | Sedimentation rate 2h (mm) | 32 | 22.4 ± 11.8 | 47 | 29.1 ± 23.8 | 0.122 |
|  | Parathyroid hormone (pg/ml) | 32 | 43.1 ± 19.3 | 47 | 61.8 ± 114.6 | 0.284 |
|  | 25 hydroxyvitamin D (µg/l) | 32 | 37.6 ± 11.3 | 47 | 37.3 ± 16.0 | 0.947 |
|  | Risk factor disease (N, %) | 10/32 | 31.3 | 22/47 | 46.8 | 0.085 |
|  | Cushing's syndrome (N, %) | 0/32 | 0.0 | 1/47 | 2.1 | >0.999 |
|  | Primary hyperparathyroidism (N, %) | 0/32 | 0.0 | 0/47 | 0.0 | - |
|  | Growth hormone deficiency (N, %) | 0/32 | 0.0 | 0/47 | 0.0 | - |
|  | Hyperthyroidism (N, %) | 0/32 | 0.0 | 1/47 | 2.1 | >0.999 |
|  | Type I diabetes mellitus (N, %) | 0/32 | 0.0 | 0/47 | 0.0 | - |
|  | Type II diabetes mellitus (N, %) | 1/32 | 3.1 | 5/47 | 10.6 | 0.231 |
|  | Celiac disease (N, %) | 1/32 | 3.1 | 1/47 | 2.1 | >0.999 |
|  | B-II gastric resection/gastrectomy (N, %) | 0/32 | 0.0 | 0/47 | 0.0 | - |
|  | Epilepsy (N, %) | 0/32 | 0.0 | 0/47 | 0.0 | - |
|  | Depression (N, %) | 2/32 | 6.3 | 4/47 | 8.5 | >0.999 |
|  | Schizophrenia (N, %) | 0/32 | 0.0 | 0/47 | 0.0 | - |
|  | Stroke (N, %) | 1/32 | 3.1 | 3/47 | 6.4 | 0.632 |
|  | Alzheimer's disease (N, %) | 0/32 | 0.0 | 0/47 | 0.0 | - |
|  | Parkinson's disease (N, %) | 0/32 | 0.0 | 0/47 | 0.0 | - |
|  | Cardiac insufficiency (N, %) | 5/32 | 15.6 | 5/47 | 10.6 | 0.736 |
|  | Renal insufficiency (N, %) | 2/32 | 6.3 | 9/47 | 19.1 | 0.103 |
|  | MGUS (N, %) | 0/32 | 0.0 | 1/47 | 2.1 | >0.999 |
|  | Risk factor drug (N, %) | 25/32 | 78.1 | 30/47 | 63.8 | 0.418 |
|  | Treatment of prostate cancer (N, %) | 0/32 | 0.0 | 1/47 | 2.1 | >0.999 |
|  | Treatment of breast cancer (N, %) | 1/32 | 3.1 | 0/47 | 0.0 | 0.427 |
|  | Treatment of epilepsy (N, %) | 0/32 | 0.0 | 0/47 | 0.0 | - |
|  | Psychotropic drugs (N, %) | 2/32 | 6.3 | 2/47 | 4.3 | >0.999 |
|  | Prednisolone (N, %) | 19/32 | 59.4 | 24/47 | 51.1 | 0.758 |
|  | Glitazone (N, %) | 1/32 | 3.1 | 1/47 | 2.1 | >0.999 |
|  | Tranquilizers (N, %) | 1/32 | 3.1 | 2/47 | 4.3 | >0.999 |
|  | Sleeping pills (N, %) | 4/32 | 12.5 | 1/47 | 2.1 | 0.157 |
|  | Analgesics (N, %) | 3/32 | 9.4 | 3/47 | 6.4 | >0.999 |
|  | Proton pump inhibitors (N, %) | 10/32 | 31.3 | 20/47 | 42.6 | 0.182 |
| **Sjögren's syndrome** | Gender (N Female, %) | 32/32 | 100.0 | 46/47 | 97.9 | >0.999 |
|  | Age (years) | 32 | 61.8 ± 9.1 | 47 | 64.0 ± 10.8 | 0.332 |
|  | Body Mass Index (kg/m2) | 32 | 26.1 ± 6.3 | 47 | 26.0 ± 5.9 | 0.947 |
|  | Size (cm) | 32 | 166.5 ± 8.0 | 47 | 164.3 ± 7.9 | 0.242 |
|  | Weight (kg) | 32 | 72.2 ± 17.1 | 47 | 70.5 ± 18.1 | 0.678 |
|  | Coffee (cups/day) | 32 | 2.0 ± 1.5 | 47 | 2.1 ± 1.6 | 0.773 |
|  | Tea (cups/day) | 32 | 0.3 ± 0.9 | 47 | 0.7 ± 1.4 | 0.112 |
|  | Alcohol (glasses/week) | 32 | 0.2 ± 0.7 | 47 | 3.2 ± 2.3 | **0.014** |
|  | Cigarettes (N/day) | 32 | 0.5 ± 2.6 | 47 | 13.0 ± 9.8 | 0.158 |
|  | Number of sports in a week | 32 | 1.8 ± 2.2 | 47 | 1.6 ± 2.1 | 0.742 |
|  | 30 min. of exercise daily (N, %) | 26/32 | 81.3 | 36/47 | 76.6 | 0.780 |
|  | Vertebrae fractures (N, %) | 2/32 | 6.3 | 13/47 | 27.7 | **0.013** |
|  | Atraumatic fractures (N, %) | 2/32 | 6.3 | 8/47 | 17.0 | 0.168 |
|  | Number of atraumatic fractures | 32 | 1.5 ± 0.7 | 47 | 1.8 ± 1.2 | 0.661 |
|  | Parents had femoral neck fracture (N, %) | 3/32 | 9.4 | 10/47 | 21.3 | 0.142 |
|  | Bone pain (NRS 0-10) | 32 | 2.5 (0 ~ 5) | 47 | 5 (1 ~ 7) | 0.078 |
|  | Laboratory values |  | | | | |
|  | Calcium (mmol/l) | 32 | 2.3 ± 0.1 | 47 | 2.3 ± 0.2 | 0.966 |
|  | CRP (mg/l) | 32 | 4.4 ± 8.8 | 47 | 2.8 ± 4.0 | 0.356 |
|  | Serum creatinine (µmol/l) | 32 | 76.5 ± 20.4 | 47 | 85.9 ± 46.8 | 0.234 |
|  | eGFR (ml/min) | 32 | 75.8 ± 16.4 | 47 | 71.8 ± 21.0 | 0.348 |
|  | Alkaline phosphatase (U/l) | 32 | 88.7 ± 39.6 | 47 | 71.9 ± 21.1 | **0.039** |
|  | Sedimentation rate 1h (mm) | 32 | 16.0 ± 15.8 | 47 | 18.1 ± 18.8 | 0.616 |
|  | Sedimentation rate 2h (mm) | 32 | 31.9 ± 19.6 | 47 | 34.0 ± 22.4 | 0.693 |
|  | Parathyroid hormone (pg/ml) | 32 | 47.3 ± 20.1 | 47 | 54.1 ± 47.5 | 0.397 |
|  | 25 hydroxyvitamin D (µg/l) | 32 | 33.7 ± 16.7 | 47 | 38.2 ± 13.8 | 0.386 |
|  | Risk factor disease (N, %) | 10/32 | 31.3 | 17/47 | 36.2 | 0.507 |
|  | Cushing's syndrome (N, %) | 1/32 | 3.1 | 1/47 | 2.1 | >0.999 |
|  | Primary hyperparathyroidism (N, %) | 0/32 | 0.0 | 0/47 | 0.0 | - |
|  | Growth hormone deficiency (N, %) | 0/32 | 0.0 | 0/47 | 0.0 | - |
|  | Hyperthyroidism (N, %) | 0/32 | 0.0 | 0/47 | 0.0 | - |
|  | Type I diabetes mellitus (N, %) | 0/32 | 0.0 | 0/47 | 0.0 | - |
|  | Type II diabetes mellitus (N, %) | 3/32 | 9.4 | 3/47 | 6.4 | 0.692 |
|  | Celiac disease (N, %) | 0/32 | 0.0 | 1/47 | 2.1 | >0.999 |
|  | B-II gastric resection/gastrectomy (N, %) | 0/32 | 0.0 | 1/47 | 2.1 | >0.999 |
|  | Epilepsy (N, %) | 1/32 | 3.1 | 5/47 | 10.6 | 0.392 |
|  | Depression (N, %) | 3/32 | 9.4 | 5/47 | 10.6 | >0.999 |
|  | Schizophrenia (N, %) | 0/32 | 0.0 | 0/47 | 0.0 | - |
|  | Stroke (N, %) | 0/32 | 0.0 | 0/47 | 0.0 | - |
|  | Alzheimer's disease (N, %) | 0/32 | 0.0 | 0/47 | 0.0 | - |
|  | Parkinson's disease (N, %) | 0/32 | 0.0 | 0/47 | 0.0 | - |
|  | Cardiac insufficiency (N, %) | 2/32 | 6.3 | 7/47 | 14.9 | 0.288 |
|  | Renal insufficiency (N, %) | 2/32 | 6.3 | 5/47 | 10.6 | 0.692 |
|  | MGUS (N, %) | 1/32 | 3.1 | 1/47 | 2.1 | >0.999 |
|  | Risk factor drug (N, %) | 16/32 | 50.0 | 33/47 | 70.2 | **0.025** |
|  | Treatment of prostate cancer (N, %) | 0/32 | 0.0 | 0/47 | 0.0 | - |
|  | Treatment of breast cancer (N, %) | 0/32 | 0.0 | 0/47 | 0.0 | - |
|  | Treatment of epilepsy (N, %) | 2/32 | 6.3 | 4/47 | 8.5 | >0.999 |
|  | Psychotropic drugs (N, %) | 1/32 | 3.1 | 6/47 | 12.8 | 0.228 |
|  | Prednisolone (N, %) | 9/32 | 28.1 | 22/47 | 46.8 | 0.055 |
|  | Glitazone (N, %) | 2/32 | 6.3 | 0/47 | 0.0 | 0.174 |
|  | Tranquilizers (N, %) | 3/32 | 9.4 | 0/47 | 0.0 | 0.071 |
|  | Sleeping pills (N, %) | 3/32 | 9.4 | 4/47 | 8.5 | >0.999 |
|  | Analgesics (N, %) | 1/32 | 3.1 | 5/47 | 10.6 | 0.392 |
|  | Proton pump inhibitors (N, %) | 13/32 | 40.6 | 23/47 | 48.9 | 0.315 |
| **Other** | Gender (N Female, %) | 21/30 | 70.0 | 15/21 | 71.4 | 0.912 |
|  | Age (years) | 30 | 63.3 ± 10.5 | 21 | 66.2 ± 10.2 | 0.328 |
|  | Body Mass Index (kg/m2) | 30 | 28.3 ± 6.6 | 21 | 26.2 ± 6.0 | 0.245 |
|  | Size (cm) | 30 | 170.4 ± 11.4 | 21 | 166.9 ± 11.0 | 0.278 |
|  | Weight (kg) | 30 | 83.1 ± 24.2 | 21 | 73.4 ± 18.6 | 0.114 |
|  | Coffee (cups/day) | 30 | 2.2 ± 2.0 | 21 | 2.4 ± 0.9 | 0.727 |
|  | Tea (cups/day) | 30 | 0.4 ± 1.2 | 21 | 0.9 ± 1.5 | 0.272 |
|  | Alcohol (glasses/week) | 30 | 1.2 ± 1.7 | 21 | 1.8 ± 1.3 | 0.530 |
|  | Cigarettes (N/day) | 30 | 2.3 ± 8.0 | 21 | 8.2 ± 3.2 | 0.054 |
|  | Number of sports in a week | 30 | 1.4 ± 2.1 | 21 | 1.0 ± 1.4 | 0.352 |
|  | 30 min. of exercise daily (N, %) | 22/30 | 73.3 | 14/21 | 66.7 | 0.689 |
|  | Vertebrae fractures (N, %) | 1/30 | 3.3 | 2/21 | 9.5 | 0.565 |
|  | Atraumatic fractures (N, %) | 1/30 | 3.3 | 3/21 | 14.3 | 0.285 |
|  | Number of atraumatic fractures | 30 | 1.0 | 21 | 1.3 ± 0.6 | - |
|  | Parents had femoral neck fracture (N, %) | 1/30 | 3.3 | 5/21 | 23.8 | **0.022** |
|  | Bone pain (NRS 0-10) | 30 | 0.5 (0 ~ 5.25) | 21 | 5 (4.5 ~ 7.5) | **0.034** |
|  | Laboratory values |  | | | | |
|  | Calcium (mmol/l) | 30 | 2.4 ± 0.1 | 21 | 2.4 ± 0.1 | 0.523 |
|  | CRP (mg/l) | 30 | 5.6 ± 10.9 | 21 | 4.5 ± 7.2 | 0.687 |
|  | Serum creatinine (µmol/l) | 30 | 75.6 ± 14.0 | 21 | 86.8 ± 37.9 | 0.222 |
|  | eGFR (ml/min) | 30 | 80.1 ± 14.6 | 21 | 72.6 ± 20.9 | 0.178 |
|  | Alkaline phosphatase (U/l) | 30 | 76.9 ± 34.1 | 21 | 73.8 ± 25.4 | 0.728 |
|  | Sedimentation rate 1h (mm) | 30 | 15.7 ± 19.5 | 21 | 21.9 ± 25.2 | 0.357 |
|  | Sedimentation rate 2h (mm) | 30 | 29.4 ± 21.9 | 21 | 36.1 ± 28.7 | 0.379 |
|  | Parathyroid hormone (pg/ml) | 30 | 33.7 ± 9.4 | 21 | 37.8 ± 18.3 | 0.380 |
|  | 25 hydroxyvitamin D (µg/l) | 30 | 31.4 ± 11.3 | 21 | 33.5 ± 16.0 | 0.692 |
|  | Risk factor disease (N, %) | 14/30 | 46.7 | 8/21 | 38.1 | 0.798 |
|  | Cushing's syndrome (N, %) | 1/30 | 3.3 | 0/21 | 0.0 | >0.999 |
|  | Primary hyperparathyroidism (N, %) | 0/30 | 0.0 | 0/21 | 0.0 | - |
|  | Growth hormone deficiency (N, %) | 0/30 | 0.0 | 0/21 | 0.0 | - |
|  | Hyperthyroidism (N, %) | 0/30 | 0.0 | 1/21 | 4.8 | 0.383 |
|  | Type I diabetes mellitus (N, %) | 0/30 | 0.0 | 1/21 | 4.8 | 0.383 |
|  | Type II diabetes mellitus (N, %) | 8/30 | 26.7 | 4/21 | 19.0 | 0.744 |
|  | Celiac disease (N, %) | 0/30 | 0.0 | 0/21 | 0.0 | - |
|  | B-II gastric resection/gastrectomy (N, %) | 0/30 | 0.0 | 0/21 | 0.0 | - |
|  | Epilepsy (N, %) | 0/30 | 0.0 | 0/21 | 0.0 | - |
|  | Depression (N, %) | 5/30 | 16.7 | 0/21 | 0.0 | 0.141 |
|  | Schizophrenia (N, %) | 0/30 | 0.0 | 0/21 | 0.0 | - |
|  | Stroke (N, %) | 0/30 | 0.0 | 0/21 | 0.0 | - |
|  | Alzheimer's disease (N, %) | 0/30 | 0.0 | 0/21 | 0.0 | - |
|  | Parkinson's disease (N, %) | 0/30 | 0.0 | 0/21 | 0.0 | - |
|  | Cardiac insufficiency (N, %) | 4/30 | 13.3 | 4/21 | 19.0 | 0.692 |
|  | Renal insufficiency (N, %) | 1/30 | 3.3 | 3/21 | 14.3 | 0.150 |
|  | MGUS (N, %) | 0/30 | 0.0 | 2/21 | 9.5 | 0.142 |
|  | Risk factor drug (N, %) | 20/30 | 66.7 | 16/21 | 76.2 | 0.164 |
|  | Treatment of prostate cancer (N, %) | 0/30 | 0.0 | 0/21 | 0.0 | - |
|  | Treatment of breast cancer (N, %) | 0/30 | 0.0 | 0/21 | 0.0 | - |
|  | Treatment of epilepsy (N, %) | 0/30 | 0.0 | 0/21 | 0.0 | - |
|  | Psychotropic drugs (N, %) | 1/30 | 3.3 | 1/21 | 4.8 | >0.999 |
|  | Prednisolone (N, %) | 15/30 | 50.0 | 12/21 | 57.1 | 0.379 |
|  | Glitazone (N, %) | 2/30 | 6.7 | 2/21 | 9.5 | 0.639 |
|  | Tranquilizers (N, %) | 2/30 | 6.7 | 0/21 | 0.0 | 0.513 |
|  | Sleeping pills (N, %) | 2/30 | 6.7 | 0/21 | 0.0 | 0.513 |
|  | Analgesics (N, %) | 2/30 | 6.7 | 3/21 | 14.3 | 0.365 |
|  | Proton pump inhibitors (N, %) | 8/30 | 26.7 | 7/21 | 33.3 | 0.466 |
| **Autoinflammation** | Gender (N Female, %) | 5/6 | 83.3 | 3/7 | 42.9 | 0.266 |
|  | Age (years) | 6 | 41.2 ± 10.2 | 7 | 61.1 ± 16.8 | **0.025** |
|  | Body Mass Index (kg/m2) | 6 | 30.4 ± 5.2 | 7 | 24.8 ± 3.9 | 0.057 |
|  | Size (cm) | 6 | 167.2 ± 6.6 | 7 | 173.1 ± 9.0 | 0.196 |
|  | Weight (kg) | 6 | 84.5 ± 11.5 | 7 | 75.0 ± 17.5 | 0.268 |
|  | Coffee (cups/day) | 6 | 2.0 ± 2.5 | 7 | 3.0 ± 1.5 | 0.479 |
|  | Tea (cups/day) | 6 | 1.1 ± 2.2 | 7 | 0.7 ± 1.0 | 0.723 |
|  | Alcohol (glasses/week) | 6 | 0.7 ± 1.2 | 7 | 2.0 ± 0.0 | 0.184 |
|  | Cigarettes (N/day) | 6 | 8.2 ± 9.6 | 7 | 3.0 ± - | - |
|  | Number of sports in a week | 6 | 0.9 ± 0.9 | 7 | 1.4 ± 1.2 | 0.466 |
|  | 30 min. of exercise daily (N, %) | 4/6 | 66.7 | 5/7 | 71.4 | >0.999 |
|  | Vertebrae fractures (N, %) | 1/6 | 16.7 | 0/7 | 0.0 | 0.462 |
|  | Atraumatic fractures (N, %) | 2/6 | 33.3 | 1/7 | 14.3 | 0.523 |
|  | Number of atraumatic fractures | 6 | 2.5 ± 0.7 | 7 | 3.0 | - |
|  | Parents had femoral neck fracture (N, %) | 1/6 | 16.7 | 1/7 | 14.3 | >0.999 |
|  | Bone pain (NRS 0-10) | 6 | 4 (1 ~ 6) | 7 | 6 (5 ~ 7) | 0.696 |
|  | Laboratory values |  | | | | |
|  | Calcium (mmol/l) | 6 | 2.3 ± 0.1 | 7 | 2.4 ± 0.0 | 0.393 |
|  | CRP (mg/l) | 6 | 1.6 ± 1.2 | 7 | 4.5 ± 8.3 | 0.393 |
|  | Serum creatinine (µmol/l) | 6 | 76.7 ± 15.1 | 7 | 88.4 ± 22.5 | 0.288 |
|  | eGFR (ml/min) | 6 | 90.3 ± 20.8 | 7 | 75.7 ± 15.3 | 0.189 |
|  | Alkaline phosphatase (U/l) | 6 | 77.8 ± 27.8 | 7 | 83.1 ± 38.0 | 0.777 |
|  | Sedimentation rate 1h (mm) | 6 | 7.5 ± 5.5 | 7 | 6.1 ± 3.9 | 0.625 |
|  | Sedimentation rate 2h (mm) | 6 | 16.0 ± 12.8 | 7 | 13.3 ± 9.3 | 0.677 |
|  | Parathyroid hormone (pg/ml) | 6 | 41.2 ± 7.9 | 7 | 58.2 ± 31.8 | 0.217 |
|  | 25 hydroxyvitamin D (µg/l) | 6 | 19.3 ± 6.5 | 7 | 43.1 ± 10.8 | **0.016** |
|  | Risk factor disease (N, %) | 2/6 | 33.3 | 1/7 | 14.3 | >0.999 |
|  | Cushing's syndrome (N, %) | 0/6 | 0.0 | 0/7 | 0.0 | - |
|  | Primary hyperparathyroidism (N, %) | 0/6 | 0.0 | 0/7 | 0.0 | - |
|  | Growth hormone deficiency (N, %) | 0/6 | 0.0 | 0/7 | 0.0 | - |
|  | Hyperthyroidism (N, %) | 0/6 | 0.0 | 0/7 | 0.0 | - |
|  | Type I diabetes mellitus (N, %) | 0/6 | 0.0 | 0/7 | 0.0 | - |
|  | Type II diabetes mellitus (N, %) | 0/6 | 0.0 | 0/7 | 0.0 | - |
|  | Celiac disease (N, %) | 0/6 | 0.0 | 0/7 | 0.0 | - |
|  | B-II gastric resection/gastrectomy (N, %) | 0/6 | 0.0 | 0/7 | 0.0 | - |
|  | Epilepsy (N, %) | 0/6 | 0.0 | 0/7 | 0.0 | - |
|  | Depression (N, %) | 2/6 | 33.3 | 0/7 | 0.0 | 0.444 |
|  | Schizophrenia (N, %) | 0/6 | 0.0 | 0/7 | 0.0 | - |
|  | Stroke (N, %) | 0/6 | 0.0 | 0/7 | 0.0 | - |
|  | Alzheimer's disease (N, %) | 0/6 | 0.0 | 0/7 | 0.0 | - |
|  | Parkinson's disease (N, %) | 0/6 | 0.0 | 0/7 | 0.0 | - |
|  | Cardiac insufficiency (N, %) | 0/6 | 0.0 | 1/7 | 14.3 | >0.999 |
|  | Renal insufficiency (N, %) | 0/6 | 0.0 | 0/7 | 0.0 | - |
|  | MGUS (N, %) | 0/6 | 0.0 | 0/7 | 0.0 | - |
|  | Risk factor drug (N, %) | 4/6 | 66.7 | 3/7 | 42.9 | >0.999 |
|  | Treatment of prostate cancer (N, %) | 0/6 | 0.0 | 0/7 | 0.0 | - |
|  | Treatment of breast cancer (N, %) | 0/6 | 0.0 | 0/7 | 0.0 | - |
|  | Treatment of epilepsy (N, %) | 0/6 | 0.0 | 0/7 | 0.0 | - |
|  | Psychotropic drugs (N, %) | 1/6 | 16.7 | 0/7 | 0.0 | >0.999 |
|  | Prednisolone (N, %) | 4/6 | 66.7 | 2/7 | 28.6 | 0.524 |
|  | Glitazone (N, %) | 0/6 | 0.0 | 0/7 | 0.0 | - |
|  | Tranquilizers (N, %) | 0/6 | 0.0 | 0/7 | 0.0 | - |
|  | Sleeping pills (N, %) | 1/6 | 16.7 | 0/7 | 0.0 | >0.999 |
|  | Analgesics (N, %) | 1/6 | 16.7 | 0/7 | 0.0 | >0.999 |
|  | Proton pump inhibitors (N, %) | 2/6 | 33.3 | 3/7 | 42.9 | >0.999 |
| **Undiagnosed** | Gender (N Female, %) | 7/12 | 58.3 | 3/3 | 100.0 | 0.505 |
|  | Age (years) | 12 | 54.8 ± 15.6 | 3 | 69.3 ± 8.1 | 0.064 |
|  | Body Mass Index (kg/m2) | 12 | 25.2 ± 2.6 | 3 | 25.0 ± 1.9 | 0.895 |
|  | Size (cm) | 12 | 173.4 ± 11.8 | 3 | 170.0 ± 6.2 | 0.515 |
|  | Weight (kg) | 12 | 76.4 ± 15.2 | 3 | 72.3 ± 8.4 | 0.555 |
|  | Coffee (cups/day) | 12 | 1.9 ± 2.3 | 3 | 2.5 ± 1.4 | 0.653 |
|  | Tea (cups/day) | 12 | 1.7 ± 2.9 | 3 | 0.0 ± 0.0 | 0.075 |
|  | Alcohol (glasses/week) | 12 | 0.4 ± 1.4 | 3 | 2.5 ± 1.4 | 0.249 |
|  | Cigarettes (N/day) | 12 | 0.6 ± 2.0 | 3 | 5.0 ± - | - |
|  | Number of sports in a week | 12 | 1.3 ± 2.1 | 3 | 2.7 ± 3.8 | 0.609 |
|  | 30 min. of exercise daily (N, %) | 11/12 | 91.7 | 2/3 | 66.7 | >0.999 |
|  | Vertebrae fractures (N, %) | 1/12 | 8.3 | 3/3 | 100.0 | **0.009** |
|  | Atraumatic fractures (N, %) | 1/12 | 8.3 | 1/3 | 33.3 | 0.371 |
|  | Number of atraumatic fractures | 12 | 3.0 | 3 | 2.0 | - |
|  | Parents had femoral neck fracture (N, %) | 1/12 | 8.3 | 0/3 | 0.0 | >0.999 |
|  | Bone pain (NRS 0-10) | 12 | 4 (2.75 ~ 5) | 3 | 5.5 (4.75 ~ 6.25) | 0.425 |
|  | Laboratory values |  | | | | |
|  | Calcium (mmol/l) | 12 | 2.4 ± 0.1 | 3 | 2.5 ± 0.1 | 0.122 |
|  | CRP (mg/l) | 12 | 3.9 ± 5.6 | 3 | 2.2 ± 1.4 | 0.348 |
|  | Serum creatinine (µmol/l) | 12 | 87.8 ± 31.8 | 3 | 82.7 ± 2.5 | 0.595 |
|  | eGFR (ml/min) | 12 | 79.3 ± 20.9 | 3 | 62.7 ± 1.5 | **0.019** |
|  | Alkaline phosphatase (U/l) | 12 | 105.7 ± 90.2 | 3 | 82.7 ± 12.5 | 0.411 |
|  | Sedimentation rate 1h (mm) | 12 | 15.0 ± 18.8 | 3 | 10.0 ± 6.1 | 0.469 |
|  | Sedimentation rate 2h (mm) | 12 | 26.6 ± 24.3 | 3 | 22.7 ± 13.3 | 0.721 |
|  | Parathyroid hormone (pg/ml) | 12 | 40.1 ± 18.0 | 3 | 44.3 ± 29.4 | 0.839 |
|  | 25 hydroxyvitamin D (µg/l) | 12 | 29.2 | 3 | 52.9 | - |
|  | Risk factor disease (N, %) | 4/12 | 33.3 | 1/3 | 33.3 | >0.999 |
|  | Cushing's syndrome (N, %) | 0/12 | 0.0 | 1/3 | 33.3 | 0.143 |
|  | Primary hyperparathyroidism (N, %) | 0/12 | 0.0 | 0/3 | 0.0 | - |
|  | Growth hormone deficiency (N, %) | 0/12 | 0.0 | 0/3 | 0.0 | - |
|  | Hyperthyroidism (N, %) | 0/12 | 0.0 | 0/3 | 0.0 | - |
|  | Type I diabetes mellitus (N, %) | 2/12 | 16.7 | 0/3 | 0.0 | >0.999 |
|  | Type II diabetes mellitus (N, %) | 1/12 | 8.3 | 1/3 | 33.3 | 0.275 |
|  | Celiac disease (N, %) | 1/12 | 8.3 | 0/3 | 0.0 | >0.999 |
|  | B-II gastric resection/gastrectomy (N, %) | 0/12 | 0.0 | 0/3 | 0.0 | - |
|  | Epilepsy (N, %) | 0/12 | 0.0 | 0/3 | 0.0 | - |
|  | Depression (N, %) | 0/12 | 0.0 | 1/3 | 33.3 | 0.143 |
|  | Schizophrenia (N, %) | 0/12 | 0.0 | 0/3 | 0.0 | - |
|  | Stroke (N, %) | 0/12 | 0.0 | 0/3 | 0.0 | - |
|  | Alzheimer's disease (N, %) | 0/12 | 0.0 | 0/3 | 0.0 | - |
|  | Parkinson's disease (N, %) | 0/12 | 0.0 | 0/3 | 0.0 | - |
|  | Cardiac insufficiency (N, %) | 0/12 | 0.0 | 1/3 | 33.3 | 0.143 |
|  | Renal insufficiency (N, %) | 0/12 | 0.0 | 0/3 | 0.0 | - |
|  | MGUS (N, %) | 0/12 | 0.0 | 0/3 | 0.0 | - |
|  | Risk factor drug (N, %) | 10/12 | 83.3 | 2/3 | 66.7 | >0.999 |
|  | Treatment of prostate cancer (N, %) | 0/12 | 0.0 | 0/3 | 0.0 | - |
|  | Treatment of breast cancer (N, %) | 0/12 | 0.0 | 0/3 | 0.0 | - |
|  | Treatment of epilepsy (N, %) | 0/12 | 0.0 | 0/3 | 0.0 | - |
|  | Psychotropic drugs (N, %) | 2/12 | 16.7 | 1/3 | 33.3 | 0.396 |
|  | Prednisolone (N, %) | 6/12 | 50.0 | 2/3 | 66.7 | 0.473 |
|  | Glitazone (N, %) | 1/12 | 8.3 | 0/3 | 0.0 | >0.999 |
|  | Tranquilizers (N, %) | 1/12 | 8.3 | 0/3 | 0.0 | >0.999 |
|  | Sleeping pills (N, %) | 0/12 | 0.0 | 0/3 | 0.0 | - |
|  | Analgesics (N, %) | 1/12 | 8.3 | 1/3 | 33.3 | 0.275 |
|  | Proton pump inhibitors (N, %) | 2/12 | 16.7 | 1/3 | 33.3 | 0.396 |

**Notes:** Low BMD was defined as described in the Methods. Values are presented as mean ± SD or n/N (%).
*Connective tissue diseases excluding SLE and SjS.
**Abbreviations:** CRP: C-reactive protein; eGFR: estimated glomerular filtration rate; NRS: numeric rating scale.

# 6 Supplementary Table 5 Association between specific immunosuppressive therapies and low BMD across all rheumatic disease entities

| **Medication** | **Patients with medication and without Low BMD** | | **Patients with medication and with Low BMD** | | **Patients with Low BMD and without medication** | | **Patients with Low BMD and with medication** | | **p** |
| --- | --- | --- | --- | --- | --- | --- | --- | --- | --- |
| **Rheumatoid arthritis (*n* = 146)** | | | | | | | | | |
| **csDMARDs** | 16/50 | 32.0% | 54/96 | 56.3% | 42/76 | 55.3% | 54/70 | 77.1% | **0.005** |
| Hydroxychloroquine | 0/50 | 0.0% | 11/96 | 11.5% | 85/135 | 63.0% | 11/11 | 100.0% | **0.016** |
| Methotrexate | 15/50 | 30.0% | 43/96 | 44.8% | 53/88 | 60.2% | 43/58 | 74.1% | 0.083 |
| Leflunomide | 1/50 | 2.0% | 2/96 | 2.1% | 94/143 | 65.7% | 2/3 | 66.7% | >0.999 |
| Sulfasalazine | 2/50 | 4.0% | 4/96 | 4.2% | 92/140 | 65.7% | 4/6 | 66.7% | >0.999 |
| **tsDMARDs** | 5/50 | 10.0% | 18/96 | 18.8% | 78/123 | 63.4% | 18/23 | 78.3% | 0.168 |
| Baricitinib | 3/50 | 6.0% | 10/96 | 10.4% | 86/133 | 64.7% | 10/13 | 76.9% | 0.543 |
| Upadacitinib | 2/50 | 4.0% | 8/96 | 8.3% | 88/136 | 64.7% | 8/10 | 80.0% | 0.495 |
| **bDMARDs** | 26/50 | 52.0% | 59/96 | 61.5% | 37/61 | 60.7% | 59/85 | 69.4% | 0.272 |
| TNF-α inhibitors | 9/50 | 18.0% | 17/96 | 17.7% | 79/120 | 65.8% | 17/26 | 65.4% | 0.965 |
| Adalimumab | 3/50 | 6.0% | 8/96 | 8.3% | 88/135 | 65.2% | 8/11 | 72.7% | 0.749 |
| Certolizumab | 2/50 | 4.0% | 4/96 | 4.2% | 92/140 | 65.7% | 4/6 | 66.7% | >0.999 |
| Etanercept | 3/50 | 6.0% | 3/96 | 3.1% | 93/140 | 66.4% | 3/6 | 50.0% | 0.412 |
| IL inhibitors | 2/50 | 4.0% | 9/96 | 9.4% | 87/135 | 64.4% | 9/11 | 81.8% | 0.332 |
| Tocilizumab | 2/50 | 4.0% | 7/96 | 7.3% | 89/137 | 65.0% | 7/9 | 77.8% | 0.719 |
| Monoclonal antibodies | 15/50 | 30.0% | 33/96 | 34.4% | 63/98 | 64.3% | 33/48 | 68.8% | 0.593 |
| Rituximab | 14/50 | 28.0% | 30/96 | 31.3% | 66/102 | 64.7% | 30/44 | 68.2% | 0.685 |
| Abatacept | 1/50 | 2.0% | 3/96 | 3.1% | 93/142 | 65.5% | 3/4 | 75.0% | >0.999 |
| **Immunoglobulins** | 0/50 | 0.0% | 3/96 | 3.1% | 93/143 | 65.0% | 3/3 | 100.0% | 0.551 |
| Any immunosuppressant taken | 41/50 | 82.0% | 91/96 | 94.8% | 5/14 | 35.7% | 91/132 | 68.9% | **0.018** |
| **Systemic lupus erythematosus (*n* = 113)** | | | | | | | | | |
| **csDMARDs** | 38/45 | 84.4% | 61/68 | 89.7% | 7/14 | 50.0% | 61/99 | 61.6% | 0.406 |
| Hydroxychloroquine | 24/45 | 53.3% | 44/68 | 64.7% | 24/45 | 53.3% | 44/68 | 64.7% | 0.227 |
| Azathioprine | 8/45 | 17.8% | 20/68 | 29.4% | 48/85 | 56.5% | 20/28 | 71.4% | 0.161 |
| Mycophenolate mofetil | 10/45 | 22.2% | 28/68 | 41.2% | 40/75 | 53.3% | 28/38 | 73.7% | **0.037** |
| Methotrexate | 3/45 | 6.7% | 7/68 | 10.3% | 61/103 | 59.2% | 7/10 | 70.0% | 0.737 |
| **bDMARDs** | 3/45 | 6.7% | 12/68 | 17.6% | 56/98 | 57.1% | 12/15 | 80.0% | 0.092 |
| Monoclonal antibodies | 3/45 | 6.7% | 12/68 | 17.6% | 56/98 | 57.1% | 12/15 | 80.0% | 0.092 |
| Belimumab | 2/45 | 4.4% | 11/68 | 16.2% | 57/100 | 57.0% | 11/13 | 84.6% | 0.056 |
| Any immunosuppressant taken | 38/45 | 84.4% | 64/68 | 94.1% | 4/11 | 36.4% | 64/102 | 62.7% | 0.111 |
| **Spondyloarthritis (*n* = 126)** | | | | | | | | | |
| **csDMARDs** | 24/68 | 35.3% | 23/58 | 39.7% | 35/79 | 44.3% | 23/47 | 48.9% | 0.614 |
| Hydroxychloroquine | 0/68 | 0.0% | 3/58 | 5.2% | 55/123 | 44.7% | 3/3 | 100.0% | 0.095 |
| Azathioprine | 2/68 | 2.9% | 1/58 | 1.7% | 57/123 | 46.3% | 1/3 | 33.3% | >0.999 |
| Methotrexate | 16/68 | 23.5% | 16/58 | 27.6% | 42/94 | 44.7% | 16/32 | 50.0% | 0.602 |
| Leflunomide | 3/68 | 4.4% | 0/58 | 0.0% | 58/123 | 47.2% | 0/3 | 0.0% | 0.249 |
| Sulfasalazine | 5/68 | 7.4% | 6/58 | 10.3% | 52/115 | 45.2% | 6/11 | 54.5% | 0.553 |
| **tsDMARDs** | 1/68 | 1.5% | 5/58 | 8.6% | 53/120 | 44.2% | 5/6 | 83.3% | 0.094 |
| Upadacitinib | 0/68 | 0.0% | 3/58 | 5.2% | 55/123 | 44.7% | 3/3 | 100.0% | 0.095 |
| Apremilast | 1/68 | 1.5% | 2/58 | 3.4% | 56/123 | 45.5% | 2/3 | 66.7% | 0.594 |
| **bDMARDs** | 33/68 | 48.5% | 38/58 | 65.5% | 20/55 | 36.4% | 38/71 | 53.5% | 0.055 |
| TNF-α inhibitors | 25/68 | 36.8% | 25/58 | 43.1% | 33/76 | 43.4% | 25/50 | 50.0% | 0.469 |
| Adalimumab | 10/68 | 14.7% | 8/58 | 13.8% | 50/108 | 46.3% | 8/18 | 44.4% | 0.884 |
| Certolizumab | 3/68 | 4.4% | 1/58 | 1.7% | 57/122 | 46.7% | 1/4 | 25.0% | 0.624 |
| Etanercept | 5/68 | 7.4% | 5/58 | 8.6% | 53/116 | 45.7% | 5/10 | 50.0% | >0.999 |
| Golimumab | 4/68 | 5.9% | 7/58 | 12.1% | 51/115 | 44.3% | 7/11 | 63.6% | 0.220 |
| Infliximab | 3/68 | 4.4% | 4/58 | 6.9% | 54/119 | 45.4% | 4/7 | 57.1% | 0.702 |
| IL inhibitors | 7/68 | 10.3% | 12/58 | 20.7% | 46/107 | 43.0% | 12/19 | 63.2% | 0.104 |
| Secukinumab | 6/68 | 8.8% | 7/58 | 12.1% | 51/113 | 45.1% | 7/13 | 53.8% | 0.551 |
| Ixekizumab | 1/68 | 1.5% | 3/58 | 5.2% | 55/122 | 45.1% | 3/4 | 75.0% | 0.333 |
| Any immunosuppressant taken | 51/68 | 75.0% | 51/58 | 87.9% | 7/24 | 29.2% | 51/102 | 50.0% | 0.065 |
| **Connective tissue disease* (*n* = 82)** | | | | | | | | | |
| **csDMARDs** | 18/35 | 51.4% | 31/47 | 66.0% | 16/33 | 48.5% | 31/49 | 63.3% | 0.185 |
| Hydroxychloroquine | 5/35 | 14.3% | 17/47 | 36.2% | 30/60 | 50.0% | 17/22 | 77.3% | **0.027** |
| Azathioprine | 8/35 | 22.9% | 7/47 | 14.9% | 40/67 | 59.7% | 7/15 | 46.7% | 0.356 |
| Mycophenolate mofetil | 4/35 | 11.4% | 5/47 | 10.6% | 42/73 | 57.5% | 5/9 | 55.6% | >0.999 |
| Methotrexate | 3/35 | 8.6% | 8/47 | 17.0% | 39/71 | 54.9% | 8/11 | 72.7% | 0.338 |
| **Calcineurin** **inhibitors** | 5/35 | 14.3% | 0/47 | 0.0% | 47/77 | 61.0% | 0/5 | 0.0% | **0.012** |
| Tacrolimus | 3/35 | 8.6% | 0/47 | 0.0% | 47/79 | 59.5% | 0/3 | 0.0% | 0.074 |
| Any immunosuppressant taken | 24/35 | 68.6% | 31/47 | 66.0% | 16/27 | 59.3% | 31/55 | 56.4% | 0.803 |
| **Vasculitis (*n* = 79)** | | | | | | | | | |
| **csDMARDs** | 14/32 | 43.8% | 27/47 | 57.4% | 20/38 | 52.6% | 27/41 | 65.9% | 0.232 |
| Hydroxychloroquine | 1/32 | 3.1% | 4/47 | 8.5% | 43/74 | 58.1% | 4/5 | 80.0% | 0.643 |
| Azathioprine | 3/32 | 9.4% | 8/47 | 17.0% | 39/68 | 57.4% | 8/11 | 72.7% | 0.510 |
| Mycophenolate mofetil | 1/32 | 3.1% | 3/47 | 6.4% | 44/75 | 58.7% | 3/4 | 75.0% | 0.643 |
| Methotrexate | 6/32 | 18.8% | 13/47 | 27.7% | 34/60 | 56.7% | 13/19 | 68.4% | 0.363 |
| Leflunomide | 3/32 | 9.4% | 3/47 | 6.4% | 44/73 | 60.3% | 3/6 | 50.0% | 0.682 |
| **bDMARDs** | 9/32 | 28.1% | 17/47 | 36.2% | 30/53 | 56.6% | 17/26 | 65.4% | 0.455 |
| TNF-α inhibitors | 2/32 | 6.3% | 1/47 | 2.1% | 46/76 | 60.5% | 1/3 | 33.3% | 0.563 |
| Infliximab | 2/32 | 6.3% | 1/47 | 2.1% | 46/76 | 60.5% | 1/3 | 33.3% | 0.563 |
| IL inhibitors | 4/32 | 12.5% | 4/47 | 8.5% | 43/71 | 60.6% | 4/8 | 50.0% | 0.708 |
| Tocilizumab | 4/32 | 12.5% | 3/47 | 6.4% | 44/72 | 61.1% | 3/7 | 42.9% | 0.432 |
| Monoclonal antibodies | 3/32 | 9.4% | 12/47 | 25.5% | 35/64 | 54.7% | 12/15 | 80.0% | 0.072 |
| Rituximab | 3/32 | 9.4% | 12/47 | 25.5% | 35/64 | 54.7% | 12/15 | 80.0% | 0.072 |
| **Immunoglobulins** | 1/32 | 3.1% | 2/47 | 4.3% | 45/76 | 59.2% | 2/3 | 66.7% | >0.999 |
| Any immunosuppressant taken | 22/32 | 68.8% | 38/47 | 80.9% | 9/19 | 47.4% | 38/60 | 63.3% | 0.217 |
| **Sjögren's syndrome (*n* = 79)** | | | | | | | | | |
| **csDMARDs** | 17/32 | 53.1% | 34/47 | 72.3% | 13/28 | 46.4% | 34/51 | 66.7% | 0.080 |
| Hydroxychloroquine | 10/32 | 31.3% | 15/47 | 31.9% | 32/54 | 59.3% | 15/25 | 60.0% | 0.950 |
| Azathioprine | 3/32 | 9.4% | 9/47 | 19.1% | 38/67 | 56.7% | 9/12 | 75.0% | 0.342 |
| Mycophenolate mofetil | 2/32 | 6.3% | 7/47 | 14.9% | 40/70 | 57.1% | 7/9 | 77.8% | 0.299 |
| Methotrexate | 3/32 | 9.4% | 6/47 | 12.8% | 41/70 | 58.6% | 6/9 | 66.7% | 0.732 |
| **bDMARDs** | 2/32 | 6.3% | 3/47 | 6.4% | 44/74 | 59.5% | 3/5 | 60.0% | >0.999 |
| Monoclonal antibodies | 2/32 | 6.3% | 3/47 | 6.4% | 44/74 | 59.5% | 3/5 | 60.0% | >0.999 |
| Rituximab | 2/32 | 6.3% | 3/47 | 6.4% | 44/74 | 59.5% | 3/5 | 60.0% | >0.999 |
| **Immunoglobulins** | 1/32 | 3.1% | 2/47 | 4.3% | 45/76 | 59.2% | 2/3 | 66.7% | >0.999 |
| Any immunosuppressant taken | 19/32 | 59.4% | 35/47 | 74.5% | 12/25 | 48.0% | 35/54 | 64.8% | 0.157 |
| **Other (*n* = 51)** | | | | | | | | | |
| **csDMARDs** | 10/30 | 33.3% | 15/21 | 71.4% | 6/26 | 23.1% | 15/25 | 60.0% | **0.007** |
| Hydroxychloroquine | 3/30 | 10.0% | 1/21 | 4.8% | 20/47 | 42.6% | 1/4 | 25.0% | 0.634 |
| Mycophenolate mofetil | 3/30 | 10.0% | 0/21 | 0.0% | 21/48 | 43.8% | 0/3 | 0.0% | 0.259 |
| Methotrexate | 4/30 | 13.3% | 14/21 | 66.7% | 7/33 | 21.2% | 14/18 | 77.8% | **<0.001** |
| **bDMARDs** | 1/30 | 3.3% | 2/21 | 9.5% | 19/48 | 39.6% | 2/3 | 66.7% | 0.561 |
| Any immunosuppressant taken | 11/30 | 36.7% | 15/21 | 71.4% | 6/25 | 24.0% | 15/26 | 57.7% | **0.015** |
| **Autoinflammation (*n* = 13)** | | | | | | | | | |
| **csDMARDs** | 1/6 | 16.7% | 3/7 | 42.9% | 4/9 | 44.4% | 3/4 | 75.0% | 0.559 |
| Methotrexate | 1/6 | 16.7% | 3/7 | 42.9% | 4/9 | 44.4% | 3/4 | 75.0% | 0.559 |
| **bDMARDs** | 4/6 | 66.7% | 3/7 | 42.9% | 4/6 | 66.7% | 3/7 | 42.9% | 0.592 |
| IL inhibitors | 3/6 | 50.0% | 3/7 | 42.9% | 4/7 | 57.1% | 3/6 | 50.0% | >0.999 |
| Anakinra | 3/6 | 50.0% | 3/7 | 42.9% | 4/7 | 57.1% | 3/6 | 50.0% | >0.999 |
| Any immunosuppressant taken | 5/6 | 83.3% | 5/7 | 71.4% | 2/3 | 66.7% | 5/10 | 50.0% | >0.999 |

**Notes:** Low BMD was defined as described in the Methods. Values are presented as mean ± SD or n/N (%).
*Connective tissue diseases excluding SLE and SjS.
**Abbreviations:** bDMARDs: biological disease-modifying antirheumatic drugs; BMI: Body Mass Index; csDMARD: conventional synthetic disease-modifying antirheumatic drug; CTD: connective tissue disease; eGFR: estimated glomerular filtration rate; tsDMARDs: targeted synthetic disease-modifying antirheumatic drugs.
